# Supplementary material for: Loss of height predicts total and cardiovascular mortality: a cohort study of northern European women
Source: BMJ Open. 2021 Aug 9;11(8):e049122. doi: 10.1136/bmjopen-2021-049122 (PMC8404445; doi:10.1136/bmjopen-2021-049122)
Supplement: Supplementary data [file bmjopen-2021-049122supp001.pdf]

Supplemental table S1. Hazard ratios[a] and 95% confidence intervals for the association between height loss and total mortality in age-stratified samples of Swedish and Danish women (n=2406).

	Total mortality			
	Model 1[b]		Model 2[c, d]	
**Pooled sample stratified by baseline age[d]**	**< 50 years**	**≥ 50 years**	**< 50 years**	**≥ 50 years**
No. of cases /censored [a]	157/1183	468/598	157/1172	467/597
Height loss (cm)	1.14 (0.98, 1.34)	1.21 (1.14, 1.29)	1.14 (0.97; 1.34)	1.16 (1.08; 1.23)
Height loss (cm), binary:				
Stable (≤2)	1 (reference)	1 (reference)	1 (reference)	1 (reference)
Major height loss (>2)	1.90 (1.04, 3.46)	1.84 (1.48, 2.31)	2.02 (1.10; 3.68)	1.70 (1.36; 2.14)

[a] Number of deaths since second height measure / censored at end of follow-up. [b] Model 1 adjusted for age at follow-up, age^2, time interval and height at baseline. [c]Model 2 adjusted for age at follow-up, age^2, time interval, height at baseline, weight at baseline, baseline smoking, ethanol intake at baseline, baseline education and leisure time physical activity at both baseline and follow-up. [d] Interaction term for continuous height loss*baseline age, p=0.9. Interaction term for binary height loss*baseline age, p=0.6.
