# Supplementary material for: Loss of height predicts total and cardiovascular mortality: a cohort study of northern European women
Source: BMJ Open. 2021 Aug 9;11(8):e049122. doi: 10.1136/bmjopen-2021-049122 (PMC8404445; doi:10.1136/bmjopen-2021-049122)
Supplement: Author's manuscript [file bmjopen-2021-049122.draft_revisions.pdf]

BMJ Open

BMJ Open is committed to open peer review. As part of this commitment we make the peer review history of every article we publish publicly available.

When an article is published we post the peer reviewers' comments and the authors' responses online. We also post the versions of the paper that were used during peer review. These are the versions that the peer review comments apply to.

The versions of the paper that follow are the versions that were submitted during the peer review process. They are not the versions of record or the final published versions. They should not be cited or distributed as the published version of this manuscript.

BMJ Open is an open access journal and the full, final, typeset and author-corrected version of record of the manuscript is available on our site with no access controls, subscription charges or pay-per-view fees (http://bmjopen.bmj.com).

If you have any questions on BMJ Open's open peer review process please email info.bmjopen@bmj.com

Loss of height in relation to total and cardiovascular mortality: a cohort study of northern European women

Journal:	*BMJ Open*
Manuscript ID	bmjopen-2021-049122
Article Type:	Original research
Date Submitted by the Author:	18-Jan-2021
Complete List of Authors:	Klingberg, Sofia; University of Gothenburg, Department of Internal Medicine and Clinical Nutrition; University of Gothenburg, School of Public Health and Community Medicine<br>Dangol, Rojina; Bispebjerg and Fredriksberg Hospitals<br>Mehlig, Kirsten; University of Gothenburg, School of Public Health and Community Medicine<br>Björkelund, Cecilia; University of Gothenburg Sahlgrenska Academy, Primary Health Care/School of Public Health and Community Medicine<br>Heitmann, Berit; Bispebjerg and Frederiksberg Hospital, The Parker Institut, Research Unit for Dietary Studies; Sydney Medical School, The Boden Institute of Obesity, Nutrition, Exercise & Eating Disorders<br>Lissner, Lauren; University of Gothenburg, School of Public Health and Community Medicine
Keywords:	EPIDEMIOLOGY, PREVENTIVE MEDICINE, PUBLIC HEALTH

SCHOLARONE™
Manuscripts

I, the Submitting Author has the right to grant and does grant on behalf of all authors of the Work (as defined in the below author licence), an exclusive licence and/or a non-exclusive licence for contributions from authors who are: i) UK Crown employees; ii) where BMJ has agreed a CC-BY licence shall apply, and/or iii) in accordance with the terms applicable for US Federal Government officers or employees acting as part of their official duties; on a worldwide, perpetual, irrevocable, royalty-free basis to BMJ Publishing Group Ltd ("BMJ") its licensees and where the relevant Journal is co-owned by BMJ to the co-owners of the Journal, to publish the Work in this journal and any other BMJ products and to exploit all rights, as set out in our licence.

The Submitting Author accepts and understands that any supply made under these terms is made by BMJ to the Submitting Author unless you are acting as an employee on behalf of your employer or a postgraduate student of an affiliated institution which is paying any applicable article publishing charge ("APC") for Open Access articles. Where the Submitting Author wishes to make the Work available on an Open Access basis (and intends to pay the relevant APC), the terms of reuse of such Open Access shall be governed by a Creative Commons licence – details of these licences and which Creative Commons licence will apply to this Work are set out in our licence referred to above.

Other than as permitted in any relevant BMJ Author's Self Archiving Policies, I confirm this Work has not been accepted for publication elsewhere, is not being considered for publication elsewhere and does not duplicate material already published. I confirm all authors consent to publication of this Work and authorise the granting of this licence.

For peer review only

Loss of height in relation to total and cardiovascular mortality: a cohort study of northern European women

Sofia Klingberg[1, 2], Kirsten Mehlig[1], Rojina Dangol[3], Cecilia Björkelund[1], Berit L. Heitmann[3,4,5,6,*] and Lauren Lissner[1,*].

[1]School of Public Health and Community Medicine, Institute of Medicine, Sahlgrenska Academy, University of Gothenburg, Gothenburg, Sweden.

[2] Department of Internal Medicine and Clinical Nutrition, Institute of Medicine, Sahlgrenska Academy, University of Gothenburg, Gothenburg, Sweden

[3] Research Unit for Dietary Studies, The Parker Institute, Bispebjerg and Fredriksberg Hospitals, The Capital Region, Denmark.

[4] Center for Clinical Research and Prevention, Bispebjerg and Fredriksberg Hospitals, The Capital Region, Denmark.

[5] The Boden Institute of Obesity, Nutrition, Exercise & Eating Disorders, Sydney Medical School, Sydney, Australia

[6] Section for General Practice, Department of Public Health, University of Copenhagen, Denmark.

*Shared last authorship

Author contributions: BH and LL initiated the project. SK and KM performed all statistical analyses. SK had main responsibility for writing the article. All authors contributed to the statistical analyses and interpretation and provided comments on the manuscript. All authors read and approved the final manuscript.

Corresponding author: Sofia Klingberg, Department of Internal Medicine and Clinical Nutrition, Institute of Medicine, Sahlgrenska Academy, University of Gothenburg, P.O Box 459, 405 30 Gothenburg, Sweden. sofia.klingberg@gu.se

For peer review only - http://bmjopen.bmj.com/site/about/guidelines.xhtml

Acknowledgements: This work was supported by grants from the Swedish Research Council (VR521-2010-2984), the Swedish Research Council for Health, working Life, and Welfare (Forte, Epilife 2006-1506), and the Swedish ALF-agreement (ALFGBG-672971 and ALFGBG-722441). The funding sources had no role in study design, collection, analysis and interpretation of data, the writing of the report, or the decision to submit the article for publication. The authors declare no conflict of interest.

Transparency statement: SK affirms that the manuscript is an honest, accurate, and transparent account of the study being reported and that no important aspects of the study have been omitted.

Keywords: height loss, longitudinal, women, mortality, cardiovascular disease

For peer review only

Abstract

Objective: To examine height changes in middle-aged northern European women in relation to overall and cause-specific mortality.

Design: Population-based cohort studies with longitudinally measured heights and register-based mortality

Setting: Sweden and Denmark

Participants: Population-based samples of 2406 Swedish and Danish women born on selected years 1908-1952, recruited to baseline examinations at ages 30-60, and re-examined 10-12 years later.

Main outcome measure: Total and CVD specific mortality during 17-19 years of follow-up after last height measure.

Results: For each 1 cm height loss during 10-12 years, the hazard ratio (HR) (95% CI) for total mortality was 1.15 (1.06 to 1.24) in Swedish women and 1.22 (1.10 to 1.36) in Danish women, independent of key covariates. Multivariable analyses revealed that low height and high leisure time physical activity at baseline were independently protective of height loss. Considering total mortality, the HR for major height loss, defined as height loss > 2 cm, were 1.76 (1.34 to 2.32) in Swedish women and 1.87 (1.33 to 2.61) in Danish women. Pooled analyses indicated that height loss was monotonically associated with an increased mortality, confirming a significant effect above 2 cm height loss. For cause-specific mortality, major height loss was associated with a HR of 2.47 (1.19 to 2.36) for stroke mortality, 2.27 (1.57 to 3.30) for total CVD mortality and 1.72 (1.29 to 2.29) for mortality due to causes other than CVD.

For peer review only - http://bmjopen.bmj.com/site/about/guidelines.xhtml

Conclusion: Height loss is a marker for excess mortality in northern European women. Specifically the hazard of CVD mortality is increased in women with height loss during middle age, and the results suggest that the strongest cause-specific endpoint may be stroke mortality. The present findings suggest attention to height loss in early- and mid-adulthood to identify women at high-risk of CVD, and that regular physical activity may prevent early onset height loss.

Strengths and limitations of the study

- The population-based sampling, the prospective designs, the standardized measurement protocols and the long durations of follow-up for endpoints through high-quality national registries are major strengths of this study.
- The number of deaths due to stroke was low and results regarding stroke should therefore be interpreted with some caution.
- Based on the observational design we cannot rule out residual confounding by unmeasured factors, such as early life exposure of physical activity and smoking, peak bone mass, diseases and medical treatments.

For peer review only

Introduction

Adult height is generally maintained from the end of puberty until the beginning of the 5th decade at which time height starts to decline (1-4). Height loss is a process caused by shrinking of vertebral discs (5), spinal compression fractures (6) and change in posture (7), accelerating from the 7th decade of life (1-4). Height loss is a predictor of both low bone mineral density (6) and clinical manifestations of osteoporotic fractures, which show a gradient across the northern-southern latitude (8).

Although height loss could be thought of as part of the normal ageing process, a rapid decline has been suggested to predict overall mortality risk in two studies of men (9, 10), one in both sexes combined (11) and a single study specifically in women (12). Height loss has also been specifically associated with deaths due to cardiovascular disease (CVD) in men (10) and sexes combined (11). However, most studies have been performed in older populations and no study has to our knowledge reported sex-specific estimates for women followed from early- to mid-adulthood. The fact that the effects of height loss in women has not been studied more thoroughly is remarkable since women tend to lose more height than men (1-4). Thus, the aim of the present study was to determine if height loss in mid-life predicts overall mortality as well as mortality due to CVD in two longitudinal, population-based samples of middle-aged Nordic women.

For peer review only - http://bmjopen.bmj.com/site/about/guidelines.xhtml

Methods

Study populations

Data from two prospective cohort studies were analyzed: the Swedish Prospective Population Study of Women in Gothenburg (PPSWG) and the female cohort of the Danish arm of the MONItoring trends and determinants of CArdiovascular disease (MONICA) study. The analyses were performed in each cohort separately and after pooling of the cohorts.

The PPSWG, initiated in 1968-1969, recruited a representative sample of 1462 women, born on specific dates in 1908, 1914, 1918, 1922 and 1930, to a health examination. The participation rate at baseline was over 90% (13). In 1980-81, a re-examination took place. A total of 1153 women were examined in PPSWG at both time-points, and were thus eligible for the present study.

The Danish MONICA study recruited 1765 randomly selected female residents of western Copenhagen born in 1922, 1932, 1942 and 1952 to a health examination in 1982-1984 (14). The baseline participation rate was 79% (14). A re-examination took place in 1994, in which 1264 women took part, constituting the eligible sample from MONICA.

The total number of Swedish and Danish women for this study was thus 2417. Exclusions of six Swedish and five Danish women due to implausible height increase of ≥ 2 cm, left a final analytical sample of 2406 women.

For peer review only - http://bmjopen.bmj.com/site/about/guidelines.xhtml

For peer review only

Participants from both study cohorts provided informed consent to participate. Since 1980, all examinations in PPSWG have been approved by the Regional Ethics Review Board in Gothenburg, in accordance with the Declaration of Helsinki (registration number T331-14 for linkage to national registries). The MONICA study was approved by the Local Ethics Committee of Copenhagen County and the Danish Data protection Office (J.nr 2015-41-3942), and was in accordance with the principles of the Helsinki Declaration.

Height and other covariates

At both examinations, height was measured with a stadiometer on subjects without shoes. Measurement of height was generally performed early in the day. Height loss was calculated as baseline height minus height at follow-up. Thus, a positive value indicated height loss.

Potential confounders (age, time between measures, and baseline measures of height, weight, smoking, leisure time physical activity (LTPA), ethanol intake and education) were selected based on their known associations with height loss and mortality. All confounders were fully harmonized between the studies and included in fully adjusted models. Time between measures was calculated as the difference in time between follow-up measure and baseline measure. Participants were weighed wearing light clothing on a calibrated scale. Information on all other covariates was collected by questionnaires. Smoking status was categorized into never, former, and current smokers. LTPA was assessed by a question placing the participants in one of four categories ranging from sedentary to vigorous activity. Participants were then classified into three categories: almost inactive (low LTPA); at least four hours of low impact physical activity per week (medium LTPA); or regular

For peer review only

physical exercise or competitive sports (high LTPA). Habitual intake of beer, wine and spirits during the last year was assessed based on which ethanol intake per day was estimated. Educational level was dichotomized into compulsory education vs. more than compulsory. In PPSWG compulsory education ranged from 6 to 7 years in the different birth cohorts, while 7 years of schooling was compulsory for all birth cohorts in MONICA. Information on menopausal status was available but not included because of the collinearity with age.

End points

Date and cause of death were assessed through national mortality registries. The Danish cohort was followed for total mortality until Oct 4th 2012 with a maximum follow-up of 19.3 years, and until 31st Dec 2010 for cause specific mortality with a maximum follow-up of 16.7 years. The Swedish cohort was followed until 2014, but follow-up was restricted to 19.3 and 16.7 years for total mortality and cause specific mortality, respectively, to harmonize with the Danish cohort. Death due to CVD was identified by ICD-8/9 codes 390 to 459 or ICD-10 codes I00-I99, and death specifically due to stroke was identified by ICD-8/9 codes 430 to 434 or ICD-10 codes I60-I64. Unspecified or uncertain stroke diagnoses in the Swedish cohort were verified through medical records (15).

For peer review only

Statistical analyses

The Cox proportional hazards model was used to investigate if height loss was associated with overall mortality as well as cause-specific mortality. Time of follow-up from last height measure until date of death or censoring was used as underlying time metric. Height loss was parameterized both as linear predictor and as binary predictor. Height loss was dichotomized at 2 cm, defining major height loss by height loss >2 cm. Restricted cubic spline regression was used to further investigate non-linear associations between height loss and hazard ratio (HR) for the outcomes. Three knots were automatically placed at: -0.7; 0.8; and 2.5 cm, with the reference set to zero (no height loss). Because of missing data for smoking, LTPA, education and/or ethanol intake 5 Danish and 8 Swedish women were excluded from models that adjusted for these covariates. The proportional hazards assumption (PHA) of the Cox model was tested by inclusion of a product term between the major height loss variable and survival time until death or censoring for the respective outcome. These tests did not indicate violation of the PHA (all $p>0.57$). Linear regression and logistic regression were used for investigation of association between covariates and height loss.

In order to investigate effect modification by baseline age, interactions between age (dichotomized at <50 vs. ≥50 years) and height loss (both linear and dichotomized) on overall mortality were investigated. To rule out influence of large height losses on the linear analyses, women with height loss ≥ 5 cm and ≥ 4 cm, respectively, were excluded in sensitivity analyses of the main outcome, total mortality.

For peer review only - http://bmjopen.bmj.com/site/about/guidelines.xhtml

All statistical analyses were performed in SAS, version 9.3 (SAS institute, Cary, NC). Results with p-values of <0.05 was considered significant (two-sided test). A HR was considered significant when the 95% confidence interval (CI) did not include 1.

Patient and public involvement

Patients or the public were not involved in this specific research project.

For peer review only

For peer review only - http://bmjopen.bmj.com/site/about/guidelines.xhtml

Results

Characteristics of the study population are presented in Table 1. At baseline, the average ages of the Swedish and Danish cohorts were 47 and 44 years, respectively and two-thirds were aged 38-52 years at baseline. In the pooled cohort, women lost on average 0.8 cm of height (range -1.9 to 14.0) over 11.4 years. During follow-up over a maximum of 19.3 years, 316 and 309 cases of all-cause mortality occurred in the Swedish and Danish cohorts, respectively. Considering cause-specific mortality over a maximum of 16.7 years, CVD was the primary cause in 157 cases, of which 37 were specifically due to stroke, while 362 cases were due to non-CVD causes (in total 519 deaths).

The first stage of the analyses examined mortality irrespective of cause. The hazard for total mortality for each cm of height loss was 1.18 (1.09 to 1.28) for the Swedish women and 1.24 (1.13 to 1.37) for the Danish women, after adjusting for age, baseline height and time between height measures (Table 2). Further adjustment for baseline weight and lifestyle factors gave similar estimates. Major height loss, defined as height loss > 2cm, was associated with 76 and 87% higher hazard for total mortality in Swedish and Danish women, respectively. Based on the similarities of the separate results, we pooled the cohorts. Pooled analyses suggested that height loss was associated with a monotonic increase in total mortality hazard (Figure 1) with a significant effect of a height loss of > 2 cm. Stratification by baseline age showed that the effect was similar in women aged <50 and ≥50 years at baseline in models with continuous height (interactions p=0.98) as well as dichotomized height (interactions p≥0.13) (Table S1).

For peer review only - http://bmjopen.bmj.com/site/about/guidelines.xhtml

For peer review only

All further analyses on cause-specific mortality were performed on the pooled sample due to the limited number of outcomes. These analyses suggested that continuous height loss was associated with total CVD mortality, stroke mortality and non-CVD mortality (Table 2). Major height loss was associated with a HR (95% CI) of 2.47 (1.19 to 5.13) for stroke mortality, 2.27 (1.57 to 3.30) for total CVD mortality and 1.72 (1.29 to 2.29) for mortality due to other causes, independent of age, time interval between height measures, baseline height, baseline weight, cohort and lifestyle factors (Table 2). Stroke mortality did, however, not account for the effect of height loss on overall mortality, since height loss also was associated with non-stroke CVD mortality (HR (95% CI) 1.85 (1.46 to 2.35)) (data not shown).

Sensitivity analyses were performed to rule out the possibility that the continuous effect of decreased height on overall mortality was driven by large decreases in only few individuals. Excluding women with a loss of height ≥5 cm (n=11) did not weaken the effect estimate of linear height on total mortality HR (95% CI) 1.22 (1.11 to 1.34). Similarly, when further excluding 14 women with a loss of ≥4 cm, the effect estimate remained at the same magnitude 1.20 (1.09 to 1.33).

Age-adjusted associations between covariates and height loss were also investigated (data not shown). Baseline height was consistently associated to greater subsequent height loss, both linear height loss (β=0.007, p=0.03) and major height loss (OR (95% CI) 1.03 (1.00 to 1.06). Aside from baseline height, the only other factor associated to height loss was LTPA. High LTPA was, compared to medium LTPA, linearly associated with lesser height loss (β=-0.15, p=0.02) while the association with major height loss was not significant (OR (95% CI)

For peer review only - http://bmjopen.bmj.com/site/about/guidelines.xhtml

0.85 (0.52 to 1.37). In contrast, low LTPA was, compared to medium LTPA, not associated with height loss (data not further shown).

Discussion

Statement of principal findings

In this study of middle-aged Nordic women, major height loss was associated with an elevated hazard of overall mortality of around 80%. Specifically, major height loss associated with CVD mortality, with more than a 2-fold risk for stroke mortality. The findings were independent of age, time between height measures, cohort, and baseline values of height, weight, education and lifestyle factors.

Strengths and limitations of the study

The main strengths of this study are its population-based sampling, the prospective designs, the standardized measurement protocols and the long durations of follow-up for endpoints. Furthermore, trained staff measured height using standardized methodology, and mortality was ascertained through high-quality national registries (16, 17), thereby limiting potential bias in both exposure and outcome. Also, differences between the cohorts in study design, age and period were minor with results for overall mortality that were strikingly similar in both samples, and results for CVD mortality that were independent of time between measures, age and cohort. However, a limitation is that deaths due to stroke were quite few, which implies that these results should be interpreted with some caution. Additionally, based on the observational design we cannot rule out residual confounding by unmeasured factors, such as early life exposure of physical activity and smoking, peak bone mass, diseases and medical treatments.

For peer review only - http://bmjopen.bmj.com/site/about/guidelines.xhtml

For peer review only

Comparison with other studies and interpretation

To the best of our knowledge, this study is the first to report results on the effect of height loss on mortality in women followed from middle age. Previous studies of men and mixed samples, including populations followed from a baseline age of 40 years, have shown height loss to associate with total mortality with risk ratios or HR between 1.45 and 3.43 (9-11). Previous studies of female populations were started at older age and their results are discordant. Hillier et al showed that in a cohort of women aged ≥65 years at baseline, losing ≥ 5 cm over 15 years was associated with an increased hazard for mortality of 45%, compared to women losing less (12). However, another study of women of the same baseline age found no significant effect of 4-year height loss of > 2 cm (9). The lack of association in that study could possibly be explained by low power due to few women classified as exposed and additionally the low number of deaths during follow-up. Compared to the above-mentioned studies, the present study shows overall mortality estimates within the range of those previously reported, indicating a consistent association between height loss and mortality in both women and men and over the adult life course. The latter was further confirmed by the congruent results in women <50 years and ≥ 50 years in the present study.

Moreover, the present study points out that height loss specifically predicts CVD mortality, whereas results from previous studies are somewhat divergent. Wannamethee et al found an increased risk of CVD mortality in men (10). This was confirmed by Masunari et al who presented results for women and men together (11). On the contrary, Auyeng et al found no association in neither men nor women (9), but yet again, the number of exposed as well

For peer review only - http://bmjopen.bmj.com/site/about/guidelines.xhtml

as cases in this study was low. To our knowledge, the novel finding in the present study showing a particular association between height loss and stroke mortality has not been reported before. Wannamethee et al investigated height loss in relation to incident major stroke events in men, but found no such association (10). Furthermore, height loss and CVD are linked, both epidemiologically and mechanistically, by interrelations between bone loss or osteoporosis and CVD. A recent systematic review and meta-analysis, found that low baseline bone mineral density (BMD), and fractures, were associated to an increased risk of developing CVD (18). The pathophysiological links between these conditions are not fully understood but presumably involve chronic inflammation and oxidative stress (19). Additionally, similarities exist in the process of bone formation and vascular calcification, including involvement of a range of bone biomarkers (19). Frailty, a clinical syndrome defined by impaired physical resources (20), is another feature linked to both osteoporosis (21) and CVD (22). Different definitions of frailty exist but one of the most commonly used is the one operationalized by Fried et al. in which frailty is defined by the occurrence of three out of five criteria: unintentional weight loss, self-reported exhaustion, weakness, slow walking speed, and low physical activity (20), but not height loss. Weakness has been shown as a predominant initial sign of frailty (23). Weakness could be attributed to sarcopenia, a muscle disease common in older adults diagnosed by low muscle function in the presence of low muscle quantity or quality (24). Sarcopenia is an age-related process, but can also stem from inflammation, malnutrition, and physical inactivity (24). Thus, height loss is not recognized in the definition of frailty but it should be highlighted that height loss shares both the feature of impaired physical resources and the etiology for sarcopenia and therefore future frailty definitions could consider height loss as a potential important criteria.

For peer review only

Implications of the findings

The results from the present study may be generalized to northern latitude Caucasian women. Within the northern latitude, British men have previously been investigated (10). Populations within the northern latitude are over-represented when it comes to osteoporotic fractures (8) and, although we have not been able to find publications on differences of height loss across latitudes, it could be hypothesized that populations within the northern latitude lose more height when aging. To gain further understanding, more studies of women as well as men from this region are warranted to improve knowledge about the relation between height loss, morbidity and mortality.

Height is a simple measure that could be taken in every clinical setting, as compared to for example measurement of BMD, which acquires advanced methodology. Despite its simplicity, height measurement is rarely included in the clinical examination at the general practitioner. Taken together, these results suggest that height loss should be recognized within primary care to facilitate actions for CVD prevention, but others also indicate height loss as an important indicator of low BMD, vertebral fractures and vitamin D deficiency (6).

Knowledge on how to prevent height loss is sparse. Pharmaceutical treatment for osteoporosis with alendronate has shown to prevent height loss in addition to improving bone mineral content (25), while supplement with calcium and vitamin D has not proven to prevent height loss (26). Concerning lifestyle, physical activity has been identified as

For peer review only - http://bmjopen.bmj.com/site/about/guidelines.xhtml

protective against height loss in post-menopausal women (27). Our results confirmed that regular physical exercise could contribute significantly to height loss prevention. Still, these results suggest that moderate activity may not be enough to prevent height loss and only one in seven women in the current cohorts were active enough to benefit from physical activity in relation to decreased height loss. More research is thus needed, not only on the consequences of height loss but also on the causes in order to facilitate prevention of height loss and associated comorbidity and mortality.

For peer review only

Conclusions

Height loss during mid-life is a risk marker for earlier mortality in northern European women. Specifically the hazard of CVD mortality is increased in women with height loss, and the results suggested that stroke mortality may be a major contributor to the total CVD association. These findings suggest the need for increased attention to height loss to identify individuals at increased CVD risk. Moreover, regular physical activity may be beneficial not only in prevention of CVD, but also in prevention of height loss and thereby further contributing to CVD prevention.

For peer review only - http://bmjopen.bmj.com/site/about/guidelines.xhtml

References

1. Cline MG, Meredith KE, Boyer JT, Burrows B. Decline of height with age in adults in a general population sample: estimating maximum height and distinguishing birth cohort effects from actual loss of stature with aging. Hum Biol. 1989;61(3):415-25.
2. Sorkin JD, Muller DC, Andres R. Longitudinal change in height of men and women: implications for interpretation of the body mass index: the Baltimore Longitudinal Study of Aging. American journal of epidemiology. 1999;150(9):969-77.
3. Peter RS, Fromm E, Klenk J, Concin H, Nagel G. Change in height, weight, and body mass index: longitudinal data from Austria. Am J Hum Biol. 2014;26(5):690-6.
4. Droyvold WB, Nilsen TI, Kruger O, Holmen TL, Krokstad S, Midthjell K, et al. Change in height, weight and body mass index: Longitudinal data from the HUNT Study in Norway. Int J Obes (Lond). 2006;30(6):935-9.
5. Pfirrmann CW, Metzdorf A, Elfering A, Hodler J, Boos N. Effect of aging and degeneration on disc volume and shape: A quantitative study in asymptomatic volunteers. Journal of orthopaedic research : official publication of the Orthopaedic Research Society. 2006;24(5):1086-94.
6. Mikula AL, Hetzel SJ, Binkley N, Anderson PA. Validity of height loss as a predictor for prevalent vertebral fractures, low bone mineral density, and vitamin D deficiency. Osteoporosis international : a journal established as result of cooperation between the European Foundation for Osteoporosis and the National Osteoporosis Foundation of the USA. 2017;28(5):1659-65.
7. Tsunoda K. Height loss caused by bent posture: a risk factor for stroke from ENT clinic - is it time to reconsider the physical examination? Acta Otolaryngol. 2011;131(10):1079-85.
8. Kanis JA, Oden A, McCloskey EV, Johansson H, Wahl DA, Cooper C, et al. A systematic review of hip fracture incidence and probability of fracture worldwide. Osteoporosis international : a journal established as result of cooperation between the European Foundation for Osteoporosis and the National Osteoporosis Foundation of the USA. 2012;23(9):2239-56.
9. Auyeung TW, Lee JS, Leung J, Kwok T, Leung PC, Woo J. Effects of height loss on morbidity and mortality in 3145 community-dwelling Chinese older women and men: a 5-year prospective study. Age Ageing. 2010;39(6):699-704.
10. Wannamethee SG, Shaper AG, Lennon L, Whincup PH. Height loss in older men: associations with total mortality and incidence of cardiovascular disease. Archives of internal medicine. 2006;166(22):2546-52.
11. Masunari N, Fujiwara S, Kasagi F, Takahashi I, Yamada M, Nakamura T. Height loss starting in middle age predicts increased mortality in the elderly. Journal of bone and mineral research : the official journal of the American Society for Bone and Mineral Research. 2012;27(1):138-45.
12. Hillier TA, Lui LY, Kado DM, LeBlanc ES, Vesco KK, Bauer DC, et al. Height loss in older women: risk of hip fracture and mortality independent of vertebral fractures. Journal of bone and mineral research : the official journal of the American Society for Bone and Mineral Research. 2012;27(1):153-9.
13. Bengtsson C, Blohme G, Hallberg L, Hallstrom T, Isaksson B, Korsan-Bengtsen K, et al. The study of women in Gothenburg 1968-1969--a population study. General design, purpose and sampling results. Acta medica Scandinavica. 1973;193(4):311-8.
14. Jorgensen T. Prevalence of gallstones in a Danish population. American journal of epidemiology. 1987;126(5):912-21.
15. Blomstrand A, Blomstrand C, Ariai N, Bengtsson C, Björkelund C. Stroke incidence and association with risk factors in women: a 32-year follow-up of the Prospective Population Study of Women in Gothenburg. BMJ Open. 2014;4(10):e005173.
16. Brooke HL, Talback M, Hornblad J, Johansson LA, Ludvigsson JF, Druid H, et al. The Swedish cause of death register. European journal of epidemiology. 2017;32(9):765-73.

For peer review only - http://bmjopen.bmj.com/site/about/guidelines.xhtml

17. Helweg-Larsen K. The Danish Register of Causes of Death. Scand J Public Health. 2011;39(7 Suppl):26-9.
18. Veronese N, Stubbs B, Crepaldi G, Solmi M, Cooper C, Harvey NC, et al. Relationship Between Low Bone Mineral Density and Fractures With Incident Cardiovascular Disease: A Systematic Review and Meta-Analysis. Journal of bone and mineral research : the official journal of the American Society for Bone and Mineral Research. 2017;32(5):1126-35.
19. Vassalle C, Mazzone A. Bone loss and vascular calcification: A bi-directional interplay? Vascul Pharmacol. 2016;86:77-86.
20. Fried LP, Tangen CM, Walston J, Newman AB, Hirsch C, Gottdiener J, et al. Frailty in older adults: evidence for a phenotype. The journals of gerontology Series A, Biological sciences and medical sciences. 2001;56(3):M146-56.
21. Li G, Thabane L, Papaioannou A, Ioannidis G, Levine MA, Adachi JD. An overview of osteoporosis and frailty in the elderly. BMC Musculoskelet Disord. 2017;18(1):46.
22. Stewart R. Cardiovascular Disease and Frailty: What Are the Mechanistic Links? Clinical chemistry. 2019;65(1):80-6.
23. Xue QL, Bandeen-Roche K, Varadhan R, Zhou J, Fried LP. Initial manifestations of frailty criteria and the development of frailty phenotype in the Women's Health and Aging Study II. The journals of gerontology Series A, Biological sciences and medical sciences. 2008;63(9):984-90.
24. Cruz-Jentoft AJ, Bahat G, Bauer J, Boirie Y, Bruyere O, Cederholm T, et al. Sarcopenia: revised European consensus on definition and diagnosis. Age Ageing. 2019;48(1):16-31.
25. Prinsloo PJ, Hosking DJ. Alendronate sodium in the management of osteoporosis. Ther Clin Risk Manag. 2006;2(3):235-49.
26. Crandall CJ, Aragaki AK, LeBoff MS, Li W, Wactawski-Wende J, Cauley JA, et al. Calcium plus vitamin D supplementation and height loss: findings from the Women's Health Initiative Calcium and Vitamin D clinical trial. Menopause. 2016;23(12):1277-86.
27. Mai X, Marshall B, Hovey KM, Sperrazza J, Wactawski-Wende J. Risk factors for 5-year prospective height loss among postmenopausal women. Menopause. 2018;25(8):883-9.

For peer review only

For peer review only - http://bmjopen.bmj.com/site/about/guidelines.xhtml

Table 1. Characteristics of the two study populations and the pooled sample.

		Swedish women (n=1147)	Danish women (n=1259)	Pooled sample (n=2406)
Age distribution at baseline (n (%))				
	30-32 years	-	358 (28.4)	358 (14.9)
	38-42 years	305 (26.6)	344 (27.3)	649 (27.0)
	46-47 years	332 (29.0)	-	332 (13.8)
	50-52 years	323 (28.2)	321 (25.5)	644 (26.8)
	54-55 years	138 (12.0)	-	138 (5.7)
	60-62 years	49 (4.3)	236 (18.8)	285 (11.9)
Age at baseline height examination (mean (sd), years)		47.1 (6.1)	44.3 (10.8)	45.6 (9.0)
Age at last height examination (mean (sd), years)		59.1 (6.1)	55.2 (10.8)	57.1 (9.1)
Height baseline (mean (sd), cm)		163.7 (5.8)	163.7 (6.1)	163.7 (6.0)
Weight baseline (mean (sd), kg)		64.3 (10.3)	63.1(10.5)	63.7 (10.4)
Height loss (mean (sd), cm)		1.09 (1.21)	0.6 (1.0)	0.84 (1.12)
Time interval (mean (sd), years)		12.0 (0.2)	10.9 (0.3)	11.4 (0.6)
Rate of height loss (mean (sd), cm/year)		0.09 (0.10)	0.06 (0.09)	0.07 (0.10)
Education: compulsory or less at baseline (N (%))		793 (69.3)	411 (32.6)	1204 (50.1)
Smoking at baseline (N (%))				
	Never	603 (52.6)	415 (33.0)	1018 (42.3)
	Former	89 (7.8)	215 (17.1)	304 (12.6)
	Current	454 (39.6)	629 (50.0)	1083 (45.0)
Leisure time physical activity at baseline (N (%))				
	Low (almost inactive)	198 (17.3)	367 (29.2)	565 (23.5)
	Medium (≥4h low impact leisure time physical activity/ week)	812 (71.0)	718 (57.3)	1530 (63.8)
	High (regular physical activity or competitive sports)	136 (11.9)	174 (13.8)	310 (12.9)
Ethanol intake (mean (sd), g/day)		8.3 (10.9)	9.8 (11.6)	9.1 (11.3)

For peer review only - http://bmjopen.bmj.com/site/about/guidelines.xhtml

For peer review only

Table 2. Hazard ratios[a] and 95% confidence intervals for the association between height loss and total mortality and cause-specific mortality in Swedish and Danish women (n=2406).

		Total mortality					
		Model 1[b]			Model 2[c]		
**Sample**		**Swedish**	**Danish**	**Pooled**	**Swedish**	**Danish**	**Pooled**
	No. of cases /censored [a]	316/831	309/950	625/1781	315/824	309/945	624/1769
Height loss (cm)		1.18 (1.09, 1.28)	1.24 (1.13, 1.37)	1.20 (1.13, 1.27)	1.15 (1.06, 1.24)	1.22 (1.10, 1.36)	1.16 (1.10, 1.24)
Height loss (cm), binary:							
	Stable (≤2)	1 (reference)	1 (reference)	1 (reference)	1 (reference)	1 (reference)	1 (reference)
	Major height loss (>2)	1.86 (1.41, 2.44)	1.90 (1.36, 2.64)	1.85 (1.50, 2.29)	1.76 (1.34, 2.32)	1.87 (1.33, 2.61)	1.78 (1.44 2.20)

		Total CVD mortality		Stroke mortality		Non-CVD mortality	
**Pooled sample**		**Model 1[b]**	**Model 2[c]**	**Model 1[b]**	**Model 2[c]**	**Model 1[b]**	**Model 2[c]**
	No. of cases /censored [a]	157/2249	157/2236	37/2369	37/2356	362/2044	362/2031
Height loss (cm)		1.28 (1.17, 1.40)	1.23 (1.13, 1.35)	1.36 (1.15, 1.61)	1.31 (1.10, 1.56)	1.18 (1.09, 1.28)	1.15 (1.06, 1.24)
Height loss (cm), binary:							
	Stable (≤2)	1 (reference)	1 (reference)	1 (reference)	1 (reference)	1 (reference)	1 (reference)
	Major height loss (>2)	2.44 (1.69, 3.53)	2.27 (1.57, 3.30)	2.81 (1.38, 5.75)	2.47 (1.19, 5.13)	1.78 (1.34, 2.36)	1.72 (1.29, 2.29)

[a] Number of deaths since second height measure / censored at end of follow-up. [b] Model 1 adjusted for age at follow-up, age^2, time interval and height at baseline. Analyses of pooled sample also adjusted for cohort (Swedish/Danish). [c]Model 2 adjusted for age at follow-up, age^2, time interval, height at baseline, weight at baseline, baseline smoking, LTPA, ethanol and education. Analyses of pooled sample also adjusted for cohort (Swedish/Danish).

For peer review only - http://bmjopen.bmj.com/site/about/guidelines.xhtml

Figure legend

Figure 1. Hazard ratio (HR) for 10-12 year decrease in height in relation to total mortality and cause-specific mortality in a pooled sample of Swedish and Danish women. Models were adjusted for age at follow-up, age^2, time interval between height measures, and baseline values of height, weight, smoking, leisure time physical activity, ethanol intake and education. Zero change in height was used as reference value for HR. Dark line represents HR. Dotted lines represent 95% CI of HR. a) Total mortality. Test for curvature p=0.28, test for overall significance of curve p<0.001, test for linearity p<0.001. b) CVD mortality. Test for curvature p=0.65, test for overall significance of curve p<0.001, test for linearity p<0.001. c) Stroke mortality. Test for curvature p=0.72, test for overall significance of curve p<0.05, test for linearity p<0.05. d) Non-CVD mortality. Test for curvature p=0.45, test for overall significance of curve p<0.01, test for linearity p<0.01

For peer review only

For peer review only - http://bmjopen.bmj.com/site/about/guidelines.xhtml

a) Total mortality

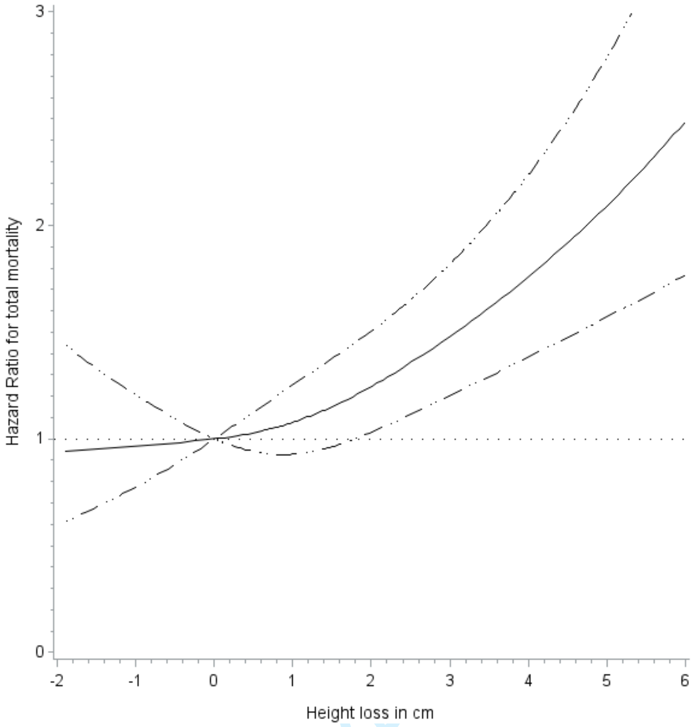

b) Total CVD mortality

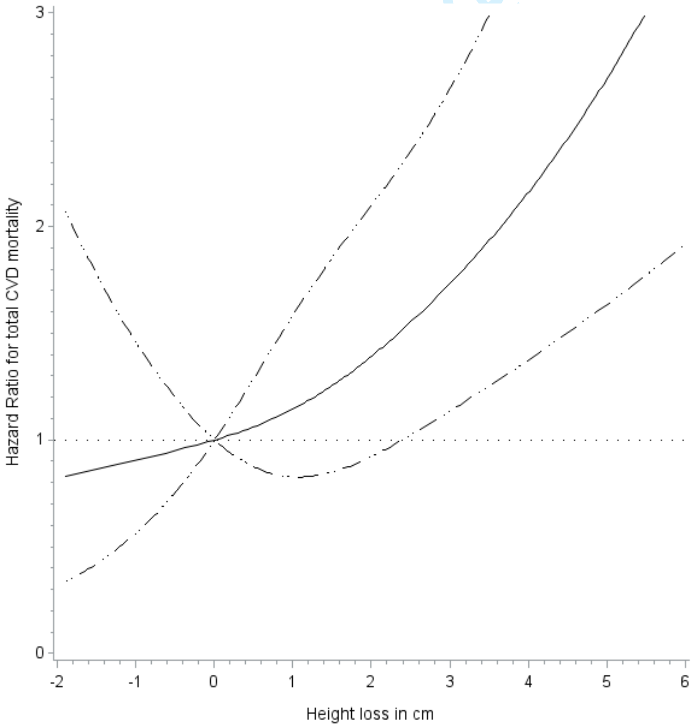

c) Stroke mortality

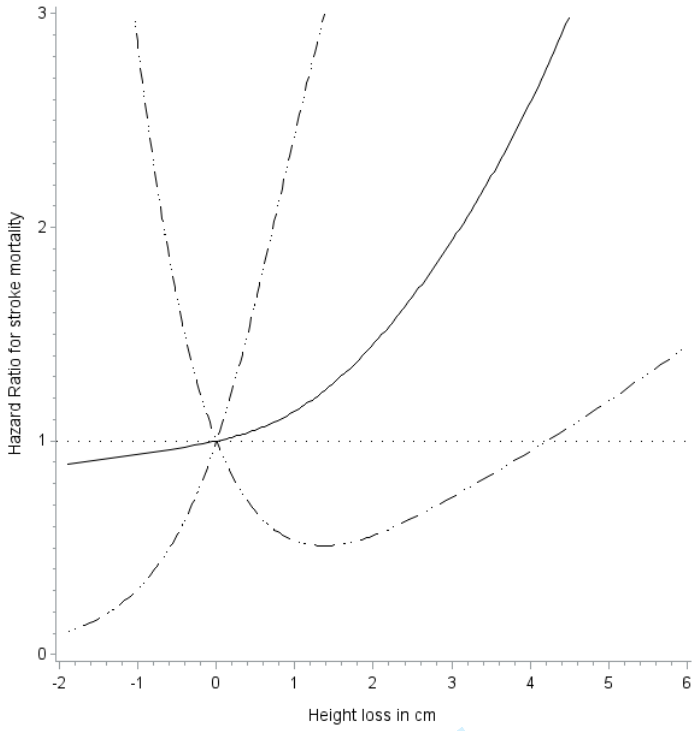

d) Non-CVD mortality

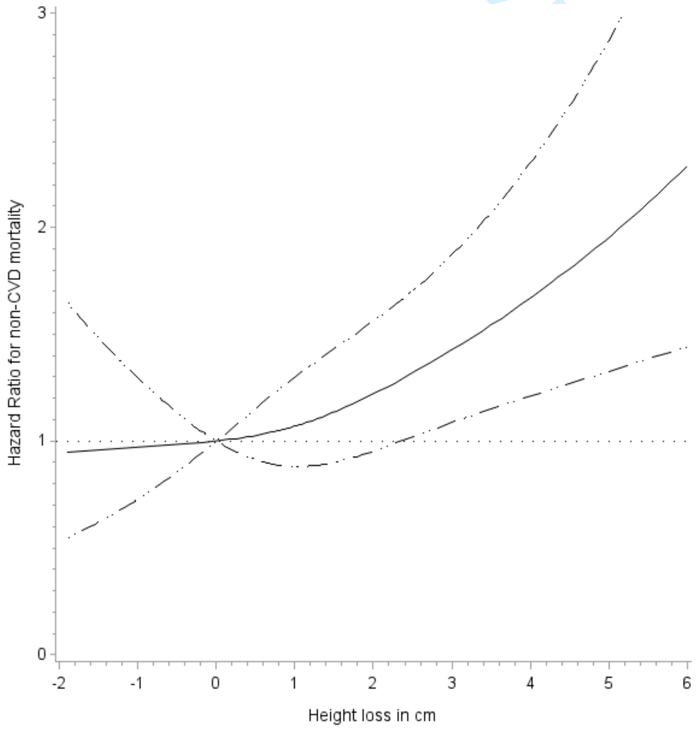

Supplemental table S1. Hazard ratios[a] and 95% confidence intervals for the association between height loss and total mortality in age-stratified samples of Swedish and Danish women (n=2406).

	**Total mortality**			
	**Model 1[b]**		**Model 2[c]**	
**Pooled sample stratified by baseline age[d]**	**< 50 years**	**≥ 50 years**	**< 50 years**	**≥ 50 years**
No. of cases /censored [a]	157/1183	468/598	157/1172	467/597
Height loss (cm)	1.14 (0.98, 1.34)	1.21 (1.14, 1.29)	1.13 (0.97, 1.32)	1.17 (1.10, 1.25)
Height loss (cm), binary:				
Stable (≤2)	1 (reference)	1 (reference)	1 (reference)	1 (reference)
Major height loss (>2)	1.90 (1.04, 3.46)	1.84 (1.48, 2.31)	1.95 (1.07, 3.55)	1.76 (1.40, 2.20)

[a] Number of deaths since second height measure / censored at end of follow-up. [b] Model 1 adjusted for age at follow-up, age^2, time interval and height at baseline. [c] Model 2 adjusted for age at follow-up, age^2, time interval, height at baseline, weight at baseline, baseline smoking, LTPA, ethanol and education. [d] Significance of interaction term for continuous height loss*baseline age was = 0.98 for both models. Significance of interaction term for binary height loss*baseline age was ≥0.13 for both models.

For peer review only

STROBE Statement—Checklist of items that should be included in reports of ***cohort studies***

	Item No	Recommendation	Reported on page #
**Title and abstract**	1	(*a*) Indicate the study's design with a commonly used term in the title or the abstract	1, 3
		(*b*) Provide in the abstract an informative and balanced summary of what was done and what was found	3-4
**Introduction**			
Background/rationale	2	Explain the scientific background and rationale for the investigation being reported	5
Objectives	3	State specific objectives, including any prespecified hypotheses	5
**Methods**			
Study design	4	Present key elements of study design early in the paper	6
Setting	5	Describe the setting, locations, and relevant dates, including periods of recruitment, exposure, follow-up, and data collection	6, 9
Participants	6	(*a*) Give the eligibility criteria, and the sources and methods of selection of participants. Describe methods of follow-up	6
		(*b*) For matched studies, give matching criteria and number of exposed and unexposed	Not applicable
Variables	7	Clearly define all outcomes, exposures, predictors, potential confounders, and effect modifiers. Give diagnostic criteria, if applicable	7-9
Data sources/ measurement	8*	For each variable of interest, give sources of data and details of methods of assessment (measurement). Describe comparability of assessment methods if there is more than one group	7-9
Bias	9	Describe any efforts to address potential sources of bias	9
Study size	10	Explain how the study size was arrived at	7
Quantitative variables	11	Explain how quantitative variables were handled in the analyses. If applicable, describe which groupings were chosen and why	7-9
Statistical methods	12	(*a*) Describe all statistical methods, including those used to control for confounding	9-10
		(*b*) Describe any methods used to examine subgroups and interactions	10
		(*c*) Explain how missing data were addressed	9
		(*d*) If applicable, explain how loss to follow-up was addressed	9
		(*e*) Describe any sensitivity analyses	10
**Results**			
Participants	13*	(a) Report numbers of individuals at each stage of study—eg numbers potentially eligible, examined for eligibility, confirmed eligible, included in the study, completing follow-up, and analysed	6
		(b) Give reasons for non-participation at each stage	6
		(c) Consider use of a flow diagram	-
Descriptive data	14*	(a) Give characteristics of study participants (eg demographic, clinical, social) and information on exposures and potential confounders	Table 1
		(b) Indicate number of participants with missing data for each variable of interest	Table 1, p. 9

For peer review only - http://bmjopen.bmj.com/site/about/guidelines.xhtml

		(c) Summarise follow-up time (eg, average and total amount)	p. 9, 11
Outcome data	15*	Report numbers of outcome events or summary measures over time	p. 11
Main results	16	(*a*) Give unadjusted estimates and, if applicable, confounder-adjusted estimates and their precision (eg, 95% confidence interval). Make clear which confounders were adjusted for and why they were included	Table 2, p. 7
		(*b*) Report category boundaries when continuous variables were categorized	p. 6, 9
		(*c*) If relevant, consider translating estimates of relative risk into absolute risk for a meaningful time period	-
Other analyses	17	Report other analyses done—eg analyses of subgroups and interactions, and sensitivity analyses	p. 11
**Discussion**			
Key results	18	Summarise key results with reference to study objectives	p. 12
Limitations	19	Discuss limitations of the study, taking into account sources of potential bias or imprecision. Discuss both direction and magnitude of any potential bias	p. 12
Interpretation	20	Give a cautious overall interpretation of results considering objectives, limitations, multiplicity of analyses, results from similar studies, and other relevant evidence	p. 13-16
Generalisability	21	Discuss the generalisability (external validity) of the study results	p. 15
**Other information**			
Funding	22	Give the source of funding and the role of the funders for the present study and, if applicable, for the original study on which the present article is based	

*Give information separately for exposed and unexposed groups.

Note: An Explanation and Elaboration article discusses each checklist item and gives methodological background and published examples of transparent reporting. The STROBE checklist is best used in conjunction with this article (freely available on the Web sites of PLoS Medicine at http://www.plosmedicine.org/, Annals of Internal Medicine at http://www.annals.org/, and Epidemiology at http://www.epidem.com/). Information on the STROBE Initiative is available at http://www.strobe-statement.org.

For peer review only

For peer review only - http://bmjopen.bmj.com/site/about/guidelines.xhtml

BMJ Open

Loss of height in relation to total and cardiovascular mortality: a cohort study of northern European women

Journal:	*BMJ Open*
Manuscript ID	bmjopen-2021-049122.R1
Article Type:	Original research
Date Submitted by the Author:	16-Apr-2021
Complete List of Authors:	Klingberg, Sofia; University of Gothenburg, Department of Internal Medicine and Clinical Nutrition; University of Gothenburg, School of Public Health and Community Medicine<br>Mehlig, Kirsten; University of Gothenburg, School of Public Health and Community Medicine<br>Dangol, Rojina; Bispebjerg and Fredriksberg Hospitals, The Parker Institute, and the Center for Clinical Research and Prevention<br>Björkelund, Cecilia; University of Gothenburg Sahlgrenska Academy, Primary Health Care/School of Public Health and Community Medicine<br>Heitmann, Berit; Bispebjerg and Fredriksberg Hospitals, The Parker Institute, and the Center for Clinical Research and Prevention; University of Copenhagen Department of Public Health, Section for General Practice<br>Lissner, Lauren; University of Gothenburg, School of Public Health and Community Medicine
**Primary Subject Heading**:	Epidemiology
Secondary Subject Heading:	Cardiovascular medicine, Public health
Keywords:	EPIDEMIOLOGY, PREVENTIVE MEDICINE, PUBLIC HEALTH, CARDIOLOGY

SCHOLARONE™
Manuscripts

I, the Submitting Author has the right to grant and does grant on behalf of all authors of the Work (as defined in the below author licence), an exclusive licence and/or a non-exclusive licence for contributions from authors who are: i) UK Crown employees; ii) where BMJ has agreed a CC-BY licence shall apply, and/or iii) in accordance with the terms applicable for US Federal Government officers or employees acting as part of their official duties; on a worldwide, perpetual, irrevocable, royalty-free basis to BMJ Publishing Group Ltd ("BMJ") its licensees and where the relevant Journal is co-owned by BMJ to the co-owners of the Journal, to publish the Work in this journal and any other BMJ products and to exploit all rights, as set out in our licence.

The Submitting Author accepts and understands that any supply made under these terms is made by BMJ to the Submitting Author unless you are acting as an employee on behalf of your employer or a postgraduate student of an affiliated institution which is paying any applicable article publishing charge ("APC") for Open Access articles. Where the Submitting Author wishes to make the Work available on an Open Access basis (and intends to pay the relevant APC), the terms of reuse of such Open Access shall be governed by a Creative Commons licence – details of these licences and which Creative Commons licence will apply to this Work are set out in our licence referred to above.

Other than as permitted in any relevant BMJ Author's Self Archiving Policies, I confirm this Work has not been accepted for publication elsewhere, is not being considered for publication elsewhere and does not duplicate material already published. I confirm all authors consent to publication of this Work and authorise the granting of this licence.

For peer review only

Loss of height in relation to total and cardiovascular mortality: a cohort study of northern European women

Sofia Klingberg[1, 2], Kirsten Mehlig[1], Rojina Dangol[3], Cecilia Björkelund[1], Berit L. Heitmann[3,4,5,*] and Lauren Lissner[1,*].

[1]School of Public Health and Community Medicine, Institute of Medicine, Sahlgrenska Academy, University of Gothenburg, Gothenburg, Sweden.

[2] Department of Internal Medicine and Clinical Nutrition, Institute of Medicine, Sahlgrenska Academy, University of Gothenburg, Gothenburg, Sweden

[3] The Parker Institute, and the Center for Clinical Research and Prevention, Bispebjerg and Fredriksberg Hospitals, The Capital Region, Denmark.

[4] Section for General Practice, Department of Public Health, University of Copenhagen, Denmark.

[5] The Boden Institute of Obesity, Nutrition, Exercise & Eating Disorders, Sydney Medical School, Sydney, Australia

*Shared last authorship

Author contributions: BLH and LL initiated the project. SK and KM performed all statistical analyses. SK had main responsibility for writing the article. SK, KM, RD, CB, BLH and LL all contributed to the statistical analyses and interpretation and provided comments on the manuscript. SK, KM, RD, CB, BLH and LL all read and approved the final manuscript.

Corresponding author: Sofia Klingberg, Department of Internal Medicine and Clinical Nutrition, Institute of Medicine, Sahlgrenska Academy, University of Gothenburg, P.O Box 459, 405 30 Gothenburg, Sweden. sofia.klingberg@gu.se

Funding statement: This work was supported by grants from the Swedish Research Council (VR521-2010-2984), the Swedish Research Council for Health, working Life, and Welfare

For peer review only - http://bmjopen.bmj.com/site/about/guidelines.xhtml

(2006-1506), and the Swedish ALF-agreement (ALFGBG-672971 and ALFGBG-722441). The funding sources had no role in study design, collection, analysis and interpretation of data, the writing of the report, or the decision to submit the article for publication. The authors declare no conflict of interest.

Transparency statement: SK affirms that the manuscript is an honest, accurate, and transparent account of the study being reported and that no important aspects of the study have been omitted.

Data availability statement: Data are available upon reasonable request. Data cannot be made publicly available for ethical and legal reasons. Public availability may compromise participant privacy, and this would not comply with Danish or Swedish legislation. Requests for data should be addressed to Professor Berit L Heitmann (Berit.Lilienthal.Heitmann@regionh.dk) and Professor Lauren Lissner (lauren.lissner@gu.se) who will provide the data access in accordance with the Danish and Swedish Data Protection Agency, respectively.

Keywords: height loss, longitudinal, women, mortality, cardiovascular disease

For peer review only

Abstract

Objective: To examine height changes in middle-aged northern European women in relation to overall and cause-specific mortality.

Design: Population-based cohort studies with longitudinally measured heights and register-based mortality.

Setting: Sweden and Denmark.

Participants: Population-based samples of 2406 Swedish and Danish women born on selected years 1908-1952, recruited to baseline examinations at ages 30-60, and re-examined 10-13 years later.

Main outcome measure: Total and CVD specific mortality during 17-19 years of follow-up after last height measure.

Results: For each 1 cm height loss during 10-13 years, the hazard ratio (HR) (95% CI) for total mortality was 1.14 (1.05 to 1.23) in Swedish women and 1.21 (1.09 to 1.35) in Danish women, independent of key covariates. Multivariable analyses revealed that low height and high leisure time physical activity at baseline were independently protective of height loss. Considering total mortality, the HR for major height loss, defined as height loss > 2 cm, were 1.74 (1.32 to 2.29) in Swedish women and 1.80 (1.27 to 2.54) in Danish women. Pooled analyses indicated that height loss was monotonically associated with an increased mortality, confirming a significant effect above 2 cm height loss. For cause-specific mortality, major height loss was associated with a HR of 2.31 (1.09 to 4.87) for stroke mortality, 2.14 (1.47 to 3.12) for total CVD mortality and 1.71 (1.28 to 2.29) for mortality due to causes other than CVD.

For peer review only

For peer review only - http://bmjopen.bmj.com/site/about/guidelines.xhtml

Conclusion: Height loss is a marker for excess mortality in northern European women. Specifically the hazard of CVD mortality is increased in women with height loss during middle age, and the results suggest that the strongest cause-specific endpoint may be stroke mortality. The present findings suggest attention to height loss in early- and mid-adulthood to identify women at high-risk of CVD, and that regular physical activity may prevent early onset height loss.

Strengths and limitations of the study

- The population-based sampling, the prospective designs, the standardized measurement protocols and the long durations of follow-up for endpoints through high-quality national registries are major strengths of this study.
- The number of deaths due to stroke was low and results regarding stroke should therefore be interpreted with some caution.
- Based on the observational design we cannot rule out residual confounding by unmeasured factors, such as early life exposure of physical activity and smoking, peak bone mass, diseases and medical treatments.

For peer review only - http://bmjopen.bmj.com/site/about/guidelines.xhtml

Introduction

Adult height is generally maintained from the end of puberty until the beginning of the 5th decade at which time height starts to decline (1-4). Height loss is a process caused by shrinking of vertebral discs (5), spinal compression fractures (6) and change in posture (7), accelerating from the 7th decade of life (1-4). Height loss is a predictor of both low bone mineral density (6) and clinical manifestations of osteoporotic fractures, which show a gradient across the northern-southern latitude (8).

For peer review only

Although height loss could be thought of as part of the normal ageing process, a rapid decline has been suggested to predict overall mortality risk in two studies of men (9, 10), one in both sexes combined (11) and a single study specifically in women (12). Height loss has also been specifically associated with deaths due to cardiovascular disease (CVD) in men (10) and sexes combined (11). However, most studies have been performed in older populations and no study has to our knowledge reported sex-specific estimates for women followed from early- to mid-adulthood. The fact that the effects of height loss in women has not been studied more thoroughly is remarkable since women tend to lose more height than men (1-4). Thus, the aim of the present study was to determine if height loss in mid-life predicts overall mortality as well as mortality due to CVD in two longitudinal, population-based samples of middle-aged Nordic women.

For peer review only - http://bmjopen.bmj.com/site/about/guidelines.xhtml

Methods

Study populations

Data from two prospective cohort studies were analyzed: the Swedish Prospective Population Study of Women in Gothenburg (PPSWG) and the female cohort of the Danish arm of the MONItoring trends and determinants of CArdiovascular disease (MONICA) study. The analyses were performed in each cohort separately and after pooling of the cohorts.

The PPSWG, initiated in 1968-1969, recruited a sample of 1462 women, born in 1908, 1914, 1918, 1922 and 1930 on specific dates that were distributed evenly across birth years. Over 90% of the invited women participated at the baseline health examination (13). In 1980-81, a re-examination took place. A total of 1153 women were examined in PPSWG at both time-points, and were thus eligible for the present study.

The Danish MONICA study recruited 1765 randomly selected female residents of western Copenhagen born in 1922, 1932, 1942 and 1952 to a health examination in 1982-1984 (14). The baseline participation rate was 79% (14). A re-examination took place in 1994, in which 1264 women took part, constituting the eligible sample from MONICA.

The total number of Swedish and Danish women for this study was thus 2417. Exclusions of six Swedish and five Danish women due to implausible height increase of ≥ 2 cm, left a final analytical sample of 2406 women.

For peer review only - http://bmjopen.bmj.com/site/about/guidelines.xhtml

Height and other covariates

At both examinations, height was measured with a stadiometer to the nearest 0.5 cm on subjects without shoes. Measurement of height was generally performed early in the day. Height loss was calculated as baseline height minus height at follow-up. Thus, a positive value indicated height loss.

Potential confounders (age, time between measures, and baseline measures of height, weight, smoking, leisure time physical activity (LTPA), ethanol intake and education) were selected based on their known associations with height loss and mortality. All confounders were fully harmonized between the studies and included in fully adjusted models. Time between measures was calculated as the difference in time between follow-up measure and baseline measure. Participants were weighed wearing light clothing on a calibrated scale. Information on all other covariates was collected by questionnaires. Smoking status was categorized into never, former, and current smokers. LTPA was assessed by a question placing the participants in one of four categories ranging from sedentary to vigorous activity (15). Participants were then classified into three categories: almost inactive (low LTPA); at least four hours of low impact physical activity per week (medium LTPA); or regular physical exercise or competitive sports (high LTPA). Habitual intake of beer, wine and spirits during the last year was assessed based on which ethanol intake per day was estimated. Educational level was dichotomized into compulsory education vs. more than compulsory. In PPSWG compulsory education ranged from 6 to 7 years in the different birth cohorts,

For peer review only - http://bmjopen.bmj.com/site/about/guidelines.xhtml

while 7 years of schooling was compulsory for all birth cohorts in MONICA. Information on menopausal status was available but not included because of the collinearity with age.

End points

Date and cause of death were assessed through national mortality registries. The Danish cohort was followed for total mortality until Oct 4th 2012 with a maximum follow-up of 19.3 years, and until 31st Dec 2010 for cause specific mortality with a maximum follow-up of 16.7 years. The Swedish cohort was followed until 2014, but follow-up was restricted to 19.3 and 16.7 years for total mortality and cause specific mortality, respectively, to harmonize with the Danish cohort. Death due to CVD was identified by ICD-8/9 codes 390 to 459 or ICD-10 codes I00-I99, and death specifically due to stroke was identified by ICD-8/9 codes 430 to 434 or ICD-10 codes I60-I64. Unspecified or uncertain stroke diagnoses in the Swedish cohort were verified through medical records (16).

For peer review only - http://bmjopen.bmj.com/site/about/guidelines.xhtml

Statistical analyses

The Cox proportional hazards model was used to investigate if height loss was associated with overall mortality as well as cause-specific mortality. Time of follow-up from last height measure until date of death or censoring was used as underlying time metric. Height loss was parameterized both as linear predictor and as binary predictor. Height loss was dichotomized at 2 cm, defining major height loss by height loss >2 cm. Restricted cubic spline regression was used to further investigate non-linear associations between height loss and hazard ratio (HR) for the outcomes. Three knots were automatically placed at: -0.7; 0.8; and 2.5 cm, with the reference set to zero (no height loss). Because of missing data for smoking, LTPA, education and/or ethanol intake 74 Danish and 12 Swedish women were excluded from models that adjusted for these covariates. The proportional hazards assumption (PHA) of the Cox model was tested by inclusion of a product term between the major height loss variable and survival time until death or censoring for the respective outcome. These tests did not indicate violation of the PHA (all p>0.64). Linear regression and logistic regression were used for investigation of association between covariates and height loss.

In order to explore effect modification by baseline age, interactions between age (dichotomized at <50 vs. ≥50 years) and height loss (both linear and dichotomized) on overall mortality were investigated. To rule out influence of large height losses on the linear analyses, women with height loss ≥ 5 cm and ≥ 4 cm, respectively, were excluded in sensitivity analyses of the main outcome, total mortality. Analyses were also repeated after exclusion of deaths within the first two years after start of follow up.

For peer review only - http://bmjopen.bmj.com/site/about/guidelines.xhtml

Interactions between cohort and height loss on cause-specific mortality were examined by inclusion of the corresponding product term in models of both linear and dichotomized height loss.

All statistical analyses were performed in SAS, version 9.3 (SAS Institute, Cary, NC). Results with p-values of <0.05 was considered significant (two-sided test). A HR was considered significant when the 95% confidence interval (CI) did not include 1.

Patient and public involvement

Patients or the public were not involved in this specific research project.

For peer review only

For peer review only - http://bmjopen.bmj.com/site/about/guidelines.xhtml

Results

Characteristics of the study population are presented in Table 1. At baseline, the average ages of the Swedish and Danish cohorts were 47 and 44 years, respectively and two-thirds were aged 38-52 years at baseline. In the pooled cohort, women lost on average 0.8 cm of height (range -1.9 to 14.0) over 11.4 years. During follow-up over a maximum of 19.3 years, 316 and 309 cases of all-cause mortality occurred in the Swedish and Danish cohorts, respectively. Considering cause-specific mortality over a maximum of 16.7 years, CVD was the primary cause in 157 cases, of which 37 were specifically due to stroke, while 362 cases were due to non-CVD causes (in total 519 deaths).

The first stage of the analyses examined mortality irrespective of cause. The hazard for total mortality for each cm of height loss was 1.18 (1.09 to 1.28) for the Swedish women and 1.24 (1.13 to 1.37) for the Danish women, after adjusting for age, baseline height and time between height measures (Table 2). Further adjustment for baseline weight and lifestyle factors gave similar estimates. Major height loss, defined as height loss > 2cm, was associated with 74 and 80% higher hazard for total mortality in Swedish and Danish women, respectively. Based on the similarities of the separate results, we pooled the cohorts. Pooled analyses suggested that height loss was associated with a monotonic increase in total mortality hazard (Figure 1) with a significant effect of a height loss of > 2 cm. Age (year) as linear factor was associated with total mortality in pooled analyses (HR (95% CI) 1.12 (1.11 to 1.14)) (data not shown). Stratification by baseline age showed that the effect was similar in women aged <50 and ≥50 years at baseline in models with continuous height (interactions p≥0.48) as well as dichotomized height (interactions p≥0.61) (Table S1).

For peer review only - http://bmjopen.bmj.com/site/about/guidelines.xhtml

For peer review only

All further analyses on cause-specific mortality were performed on the pooled sample due to the limited number of outcomes. These analyses suggested that continuous height loss was associated with total CVD mortality, stroke mortality and non-CVD mortality (Table 2). Major height loss was associated with a HR (95% CI) of 2.31 (1.09 to 4.87) for stroke mortality, 2.14 (1.47 to 3.12) for total CVD mortality and 1.71 (1.28 to 2.29) for mortality due to other causes, independent of age, time interval between height measures, baseline height, baseline weight, cohort and lifestyle factors (Table 2). Stroke mortality did, however, not account for the effect of height loss on overall mortality, since height loss also was associated with non-stroke CVD mortality (HR (95% CI) 1.82 (1.43 to 2.32) (data not shown). No significant interactions were found between height loss and cohort on cause-specific mortality (data not shown).

Sensitivity analyses were performed to rule out the possibility that the continuous effect of decreased height on overall mortality was driven by large decreases in only few individuals. Excluding women with a loss of height ≥5 cm (n=11) did not weaken the effect estimate of linear height on total mortality HR (95% CI) 1.21 (1.10 to 1.34). Similarly, when further excluding 14 women with a loss of ≥4 cm, the effect estimate remained at the same magnitude 1.19 (1.08 to 1.32). Excluding deaths occurring during the first two years of follow up (21 cases of total mortality, 6 cases of CVD mortality and 15 cases of non-CVD mortality) did not alter the results (data not shown).

For peer review only - http://bmjopen.bmj.com/site/about/guidelines.xhtml

Age-adjusted associations between covariates and height loss were also investigated (data not shown). Baseline height was consistently associated to greater subsequent height loss, both linear height loss (β=0.007, p=0.03) and major height loss (OR (95% CI) 1.03 (1.00 to 1.06). Aside from baseline height, the only other factor associated to height loss was baseline LTPA. High baseline LTPA was, compared to medium baseline LTPA, linearly associated with lesser height loss (β=-0.15, p=0.02) while the association with major height loss was not significant (OR (95% CI) 0.85 (0.52 to 1.37). In contrast, low baseline LTPA was, compared to medium baseline LTPA, not associated with height loss (data not further shown).

Discussion

Statement of principal findings

In this study of middle-aged Nordic women, major height loss was associated with an elevated hazard of overall mortality of around 80%. Specifically, major height loss associated with CVD mortality, with more than a 2-fold risk for stroke mortality. The findings were independent of age, time between height measures, cohort, and baseline values of height, weight, education and lifestyle factors.

Strengths and limitations of the study

The main strengths of this study are its population-based sampling, the prospective designs, the standardized measurement protocols and the long durations of follow-up for endpoints. Furthermore, trained staff measured height using standardized methodology, and mortality was ascertained through high-quality national registries (17, 18), thereby limiting potential bias in both exposure and outcome. Also, differences between the cohorts in study design,

For peer review only

For peer review only - http://bmjopen.bmj.com/site/about/guidelines.xhtml

age and period were minor with results for overall mortality that were strikingly similar in both samples, and results for CVD mortality that were independent of time between measures, age and cohort. However, a limitation is that deaths due to stroke were quite few, which implies that these results should be interpreted with some caution. Additionally, based on the observational design we cannot rule out residual confounding by unmeasured factors, such as early life exposure of physical activity and smoking, peak bone mass, diseases and medical treatments. Based on the fact that bone health is different depending on ethnicity it is important to take this factor into account when interpreting the results and generalizing the results. In Monica, subjects with non-Danish origin were not included (14), and in the Swedish cohort only 0.3 % were born outside Europe (13). Hence, it can be concluded that the vast majority of participants were Caucasians.

Comparison with other studies and interpretation

To the best of our knowledge, this study is the first to report results on the effect of height loss on mortality in women followed from middle age. Previous studies of men and mixed samples, including populations followed from a baseline age of 40 years, have shown height loss to associate with total mortality with risk ratios or HR between 1.45 and 3.43 (9-11). Previous studies of female populations were started at older age and their results are discordant. Hillier et al showed that in a cohort of women aged ≥65 years at baseline, losing ≥ 5 cm over 15 years was associated with an increased hazard for mortality of 45%, compared to women losing less (12). However, another study of women of the same baseline age found no significant effect of 4-year height loss of > 2 cm (9). The lack of association in that study could possibly be explained by low power due to few women classified as exposed and additionally the low number of deaths during follow-up.

For peer review only - http://bmjopen.bmj.com/site/about/guidelines.xhtml

Compared to the above-mentioned studies, the present study shows overall mortality estimates within the range of those previously reported, indicating a consistent association between height loss and mortality in both women and men and over the adult life course. The latter was further confirmed by the congruent results in women <50 years and ≥ 50 years in the present study.

Moreover, the present study points out that height loss specifically predicts CVD mortality, whereas results from previous studies are somewhat divergent. Wannamethee et al found an increased risk of CVD mortality in men (10). This was confirmed by Masunari et al who presented results for women and men together (11). On the contrary, Auyeng et al found no association in neither men nor women (9), but yet again, the number of exposed as well as cases in this study was low. To our knowledge, the novel finding in the present study showing a particular association between height loss and stroke mortality has not been reported before. Wannamethee et al investigated height loss in relation to incident major stroke events in men, but found no such association (10). Furthermore, height loss and CVD are linked, both epidemiologically and mechanistically, by interrelations between bone loss or osteoporosis and CVD. A recent systematic review and meta-analysis, found that low baseline bone mineral density (BMD), and fractures, were associated to an increased risk of developing CVD (19). The pathophysiological links between these conditions are not fully understood but presumably involve chronic inflammation and oxidative stress (20). Additionally, similarities exist in the process of bone formation and vascular calcification, including involvement of a range of bone biomarkers (20). Frailty, a clinical syndrome defined by impaired physical resources (21), is another feature linked to both osteoporosis

For peer review only - http://bmjopen.bmj.com/site/about/guidelines.xhtml

(22) and CVD (23). Different definitions of frailty exist but one of the most commonly used is the one operationalized by Fried et al. in which frailty is defined by the occurrence of three out of five criteria: unintentional weight loss, self-reported exhaustion, weakness, slow walking speed, and low physical activity (21), but not height loss. Weakness has been shown as a predominant initial sign of frailty (24). Weakness could be attributed to sarcopenia, a muscle disease common in older adults diagnosed by low muscle function in the presence of low muscle quantity or quality (25). Sarcopenia is an age-related process, but can also stem from inflammation, malnutrition, and physical inactivity (25). Thus, height loss is not recognized in the definition of frailty but it should be highlighted that height loss shares both the feature of impaired physical resources and the etiology for sarcopenia and therefore future frailty definitions could consider height loss as a potential important criteria.

Implications of the findings

The results from the present study may be generalized to northern latitude Caucasian women. Within the northern latitude, British men have previously been investigated (10). Populations within the northern latitude are over-represented when it comes to osteoporotic fractures (8) and, although we have not been able to find publications on differences of height loss across latitudes, it could be hypothesized that populations within the northern latitude lose more height when aging. To gain further understanding, more studies of women as well as men from this region are warranted to improve knowledge about the relation between height loss, morbidity and mortality.

For peer review only

For peer review only - http://bmjopen.bmj.com/site/about/guidelines.xhtml

Height is a simple measure that could be taken in every clinical setting, as compared to for example measurement of BMD, which acquires advanced methodology. Despite its simplicity, height measurement is rarely included in the clinical examination at the general practitioner. Taken together, these results suggest that height loss should be recognized within primary care to facilitate actions for CVD prevention, but others also indicate height loss as an important indicator of low BMD, vertebral fractures and vitamin D deficiency (6).

For peer review only

Knowledge on how to prevent height loss is sparse. Pharmaceutical treatment for osteoporosis with alendronate has shown to prevent height loss in addition to improving bone mineral content (26), while supplement with calcium and vitamin D has not proven to prevent height loss (27). Concerning lifestyle, physical activity has been identified as protective against height loss in post-menopausal women (28). Our results confirmed that regular physical exercise could contribute significantly to height loss prevention. Still, these results suggest that moderate activity may not be enough to prevent height loss and only one in seven women in the current cohorts were active enough to benefit from physical activity in relation to decreased height loss. More research is thus needed, not only on the consequences of height loss but also on the causes in order to facilitate prevention of height loss and associated comorbidity and mortality.

Conclusions

Height loss during mid-life is a risk marker for earlier mortality in northern European women. Specifically the hazard of CVD mortality is increased in women with height loss, and

For peer review only - http://bmjopen.bmj.com/site/about/guidelines.xhtml

the results suggested that stroke mortality may be a major contributor to the total CVD association. These findings suggest the need for increased attention to height loss to identify individuals at increased CVD risk. Moreover, regular physical activity may be beneficial not only in prevention of CVD, but also in prevention of height loss and thereby further contributing to CVD prevention.

Ethical approval statement

Participants from both study cohorts provided informed consent to participate. Since 1980, all examinations in PPSWG have been approved by the Regional Ethics Review Board in Gothenburg, in accordance with the Declaration of Helsinki (registration number T331-14 for linkage to national registries). The MONICA study was approved by the Local Ethics Committee of Copenhagen County and the Danish Data protection Office (J.nr 2015-41-3942), and was in accordance with the principles of the Helsinki Declaration.

For peer review only

For peer review only - http://bmjopen.bmj.com/site/about/guidelines.xhtml

References

1. Cline MG, Meredith KE, Boyer JT, Burrows B. Decline of height with age in adults in a general population sample: estimating maximum height and distinguishing birth cohort effects from actual loss of stature with aging. Hum Biol. 1989;61(3):415-25.
2. Sorkin JD, Muller DC, Andres R. Longitudinal change in height of men and women: implications for interpretation of the body mass index: the Baltimore Longitudinal Study of Aging. American journal of epidemiology. 1999;150(9):969-77.
3. Peter RS, Fromm E, Klenk J, Concin H, Nagel G. Change in height, weight, and body mass index: longitudinal data from Austria. Am J Hum Biol. 2014;26(5):690-6.
4. Droyvold WB, Nilsen TI, Kruger O, Holmen TL, Krokstad S, Midthjell K, et al. Change in height, weight and body mass index: Longitudinal data from the HUNT Study in Norway. Int J Obes (Lond). 2006;30(6):935-9.
5. Pfirrmann CW, Metzdorf A, Elfering A, Hodler J, Boos N. Effect of aging and degeneration on disc volume and shape: A quantitative study in asymptomatic volunteers. Journal of orthopaedic research : official publication of the Orthopaedic Research Society. 2006;24(5):1086-94.
6. Mikula AL, Hetzel SJ, Binkley N, Anderson PA. Validity of height loss as a predictor for prevalent vertebral fractures, low bone mineral density, and vitamin D deficiency. Osteoporosis international : a journal established as result of cooperation between the European Foundation for Osteoporosis and the National Osteoporosis Foundation of the USA. 2017;28(5):1659-65.
7. Tsunoda K, Research Group on the Relationship of Bent P, Stroke NHO. Height loss caused by bent posture: a risk factor for stroke from ENT clinic - is it time to reconsider the physical examination? Acta Otolaryngol. 2011;131(10):1079-85.
8. Kanis JA, Oden A, McCloskey EV, Johansson H, Wahl DA, Cooper C, et al. A systematic review of hip fracture incidence and probability of fracture worldwide. Osteoporosis international : a journal established as result of cooperation between the European Foundation for Osteoporosis and the National Osteoporosis Foundation of the USA. 2012;23(9):2239-56.
9. Auyeung TW, Lee JS, Leung J, Kwok T, Leung PC, Woo J. Effects of height loss on morbidity and mortality in 3145 community-dwelling Chinese older women and men: a 5-year prospective study. Age Ageing. 2010;39(6):699-704.
10. Wannamethee SG, Shaper AG, Lennon L, Whincup PH. Height loss in older men: associations with total mortality and incidence of cardiovascular disease. Archives of internal medicine. 2006;166(22):2546-52.
11. Masunari N, Fujiwara S, Kasagi F, Takahashi I, Yamada M, Nakamura T. Height loss starting in middle age predicts increased mortality in the elderly. Journal of bone and mineral research : the official journal of the American Society for Bone and Mineral Research. 2012;27(1):138-45.
12. Hillier TA, Lui LY, Kado DM, LeBlanc ES, Vesco KK, Bauer DC, et al. Height loss in older women: risk of hip fracture and mortality independent of vertebral fractures. Journal of bone and mineral research : the official journal of the American Society for Bone and Mineral Research. 2012;27(1):153-9.
13. Bengtsson C, Blohme G, Hallberg L, Hällström T, Isaksson B, Korsan-Bengtsen K, et al. The study of women in Gothenburg 1968-1969--a population study. General design, purpose and sampling results. Acta medica Scandinavica. 1973;193(4):311-8.
14. Jorgensen T. Prevalence of gallstones in a Danish population. American journal of epidemiology. 1987;126(5):912-21.
15. Grimby G, Börjesson M, Jonsdottir IH, Schnohr P, Thelle DS, Saltin B. The "Saltin-Grimby Physical Activity Level Scale" and its application to health research. Scandinavian journal of medicine & science in sports. 2015;25 Suppl 4:119-25.

For peer review only

For peer review only - http://bmjopen.bmj.com/site/about/guidelines.xhtml

16. Blomstrand A, Blomstrand C, Ariai N, Bengtsson C, Björkelund C. Stroke incidence and association with risk factors in women: a 32-year follow-up of the Prospective Population Study of Women in Gothenburg. BMJ Open. 2014;4(10):e005173.
17. Brooke HL, Talbäck M, Hörnblad J, Johansson LA, Ludvigsson JF, Druid H, et al. The Swedish cause of death register. European journal of epidemiology. 2017;32(9):765-73.
18. Helweg-Larsen K. The Danish Register of Causes of Death. Scand J Public Health. 2011;39(7 Suppl):26-9.
19. Veronese N, Stubbs B, Crepaldi G, Solmi M, Cooper C, Harvey NC, et al. Relationship Between Low Bone Mineral Density and Fractures With Incident Cardiovascular Disease: A Systematic Review and Meta-Analysis. Journal of bone and mineral research : the official journal of the American Society for Bone and Mineral Research. 2017;32(5):1126-35.
20. Vassalle C, Mazzone A. Bone loss and vascular calcification: A bi-directional interplay? Vascul Pharmacol. 2016;86:77-86.
21. Fried LP, Tangen CM, Walston J, Newman AB, Hirsch C, Gottdiener J, et al. Frailty in older adults: evidence for a phenotype. The journals of gerontology Series A, Biological sciences and medical sciences. 2001;56(3):M146-56.
22. Li G, Thabane L, Papaioannou A, Ioannidis G, Levine MA, Adachi JD. An overview of osteoporosis and frailty in the elderly. BMC Musculoskelet Disord. 2017;18(1):46.
23. Stewart R. Cardiovascular Disease and Frailty: What Are the Mechanistic Links? Clinical chemistry. 2019;65(1):80-6.
24. Xue QL, Bandeen-Roche K, Varadhan R, Zhou J, Fried LP. Initial manifestations of frailty criteria and the development of frailty phenotype in the Women's Health and Aging Study II. The journals of gerontology Series A, Biological sciences and medical sciences. 2008;63(9):984-90.
25. Cruz-Jentoft AJ, Bahat G, Bauer J, Boirie Y, Bruyere O, Cederholm T, et al. Sarcopenia: revised European consensus on definition and diagnosis. Age Ageing. 2019;48(1):16-31.
26. Prinsloo PJ, Hosking DJ. Alendronate sodium in the management of osteoporosis. Ther Clin Risk Manag. 2006;2(3):235-49.
27. Crandall CJ, Aragaki AK, LeBoff MS, Li W, Wactawski-Wende J, Cauley JA, et al. Calcium plus vitamin D supplementation and height loss: findings from the Women's Health Initiative Calcium and Vitamin D clinical trial. Menopause. 2016;23(12):1277-86.
28. Mai X, Marshall B, Hovey KM, Sperrazza J, Wactawski-Wende J. Risk factors for 5-year prospective height loss among postmenopausal women. Menopause. 2018;25(8):883-9.

For peer review only

For peer review only - http://bmjopen.bmj.com/site/about/guidelines.xhtml

Table 1. Characteristics of the two study populations and the pooled sample.

		Swedish women (n=1147)	Danish women (n=1259)	Pooled sample (n=2406)
Age distribution at baseline (n (%))				
	30-32 years	-	358 (28.4)	358 (14.9)
	38-42 years	305 (26.6)	344 (27.3)	649 (27.0)
	46-47 years	332 (29.0)	-	332 (13.8)
	50-52 years	323 (28.2)	321 (25.5)	644 (26.8)
	54-55 years	138 (12.0)	-	138 (5.7)
	60-62 years	49 (4.3)	236 (18.8)	285 (11.9)
Age at baseline height examination (mean (sd), years)		47.1 (6.1)	44.3 (10.8)	45.6 (9.0)
Age at last height examination (mean (sd), years)		59.1 (6.1)	55.2 (10.8)	57.1 (9.1)
Height baseline (mean (sd), cm)		163.7 (5.8)	163.7 (6.1)	163.7 (6.0)
Weight baseline (mean (sd), kg)		64.3 (10.3)	63.1(10.5)	63.7 (10.4)
Height loss (mean (sd), cm)		1.09 (1.21)	0.6 (1.0)	0.84 (1.12)
Time interval (mean (sd), years)		12.0 (0.2)	10.9 (0.3)	11.4 (0.6)
Rate of height loss (mean (sd), cm/year)		0.09 (0.10)	0.06 (0.09)	0.07 (0.10)
Education: compulsory or less at baseline (N (%))		793 (69.3)	411 (32.6)	1204 (50.1)
Smoking at baseline (N (%))				
	Never	603 (52.6)	415 (33.0)	1018 (42.3)
	Former	89 (7.8)	215 (17.1)	304 (12.6)
	Current	454 (39.6)	629 (50.0)	1083 (45.0)
Leisure time physical activity at baseline (N (%))				
	Low (almost inactive)	198 (17.3)	367 (29.2)	565 (23.5)
	Medium (≥4h low impact leisure time physical activity/ week)	812 (71.0)	718 (57.3)	1530 (63.8)
	High (regular physical activity or competitive sports)	136 (11.9)	174 (13.8)	310 (12.9)
Leisure time physical activity at follow up (n (%))				
	Low (almost inactive)	335 (29.2)	311 (26.1)	646 (27.6)

For peer review only - http://bmjopen.bmj.com/site/about/guidelines.xhtml

	Medium (≥4h low impact leisure time physical activity/ week)	582 (51.0)	735 (61.8)	1317 (56.5)
	High (regular physical activity or competitive sports)	230 (20.1)	146 (12.3)	376 (16.1)
Ethanol intake (mean (sd), g/day)		8.3 (10.9)	9.8 (11.6)	9.1 (11.3)

Table 2. Hazard ratios[a] and 95% confidence intervals for the association between height loss and total mortality and cause-specific mortality in Swedish and Danish women (n=2406).

		Total mortality					
		Model 1[b]			Model 2[c]		
**Sample**		**Swedish**	**Danish**	**Pooled**	**Swedish**	**Danish**	**Pooled**
	No. of cases /censored [a]	316/831	309/950	625/1781	314/821	292/893	606/1714
Height loss (cm)		1.18 (1.09, 1.28)	1.24 (1.13, 1.37)	1.20 (1.13, 1.27)	1.14 (1.05; 1.23)	1.21 (1.09; 1.35)	1.15 (1.09; 1.23)
Height loss (cm), binary:							
	Stable (≤2)	1 (reference)	1 (reference)	1 (reference)	1 (reference)	1 (reference)	1 (reference)
	Major height loss (>2)	1.86 (1.41, 2.44)	1.90 (1.36, 2.64)	1.85 (1.50, 2.29)	1.74 (1.32; 2.29)	1.80 (1.27; 2.54)	1.74 (1.41; 2.16)
		**Total CVD mortality**		**Stroke mortality**		**Non-CVD mortality**	
**Pooled sample**		**Model 1[b]**	**Model 2[c]**	**Model 1[b]**	**Model 2[c]**	**Model 1[b]**	**Model 2[c]**
	No. of cases /censored [a]	157/2249	156/2164	37/2369	36/2284	362/2044	347/1973
Height loss (cm)		1.28 (1.17, 1.40)	1.21 (1.10; 1.32)	1.36 (1.15, 1.61)	1.30 (1.09; 1.55)	1.18 (1.09, 1.28)	1.14 (1.05; 1.24)
Height loss (cm), binary:							
	Stable (≤2)	1 (reference)	1 (reference)	1 (reference)	1 (reference)	1 (reference)	1 (reference)
	Major height loss (>2)	2.44 (1.69, 3.53)	2.14 (1.47; 3.12)	2.81 (1.38, 5.75)	2.31 (1.09; 4.87)	1.78 (1.34, 2.36)	1.71 (1.28; 2.29)

[a] Number of deaths since second height measure / censored at end of follow-up. [b] Model 1 adjusted for age at follow-up, age^2, time interval and height at baseline. Analyses of pooled sample also adjusted for cohort (Swedish/Danish). [c] Model 2 adjusted for age at follow-up, age^2, time interval, height at baseline, weight at baseline, baseline smoking, ethanol intake at baseline, baseline education and leisure time physical activity at both baseline and follow-up. Analyses of pooled sample also adjusted for cohort (Swedish/Danish).

For peer review only - http://bmjopen.bmj.com/site/about/guidelines.xhtml

For peer review only

Figure legend

Figure 1. Hazard ratio (HR) for 10-12 year decrease in height in relation to total mortality and cause-specific mortality in a pooled sample of Swedish and Danish women. Models were adjusted for age at follow-up, age^2, time interval between height measures, and height at baseline, weight at baseline, baseline smoking, ethanol intake at baseline, baseline education and leisure time physical activity at both baseline and follow-up. Zero change in height was used as reference value for HR. Dark line represents HR. Dotted lines represent 95% CI of HR. a) Total mortality. Test for curvature p=0.28, test for overall significance of curve p<0.001, test for linearity p<0.001. b) CVD mortality. Test for curvature p=0.72, test for overall significance of curve p<0.01, test for linearity p<0.001. c) Stroke mortality. Test for curvature p=0.74, test for overall significance of curve p=0.06, test for linearity p<0.05. d) Non-CVD mortality. Test for curvature p=0.39, test for overall significance of curve p<0.05, test for linearity p<0.01.

For peer review only

For peer review only - http://bmjopen.bmj.com/site/about/guidelines.xhtml

a) Total mortality

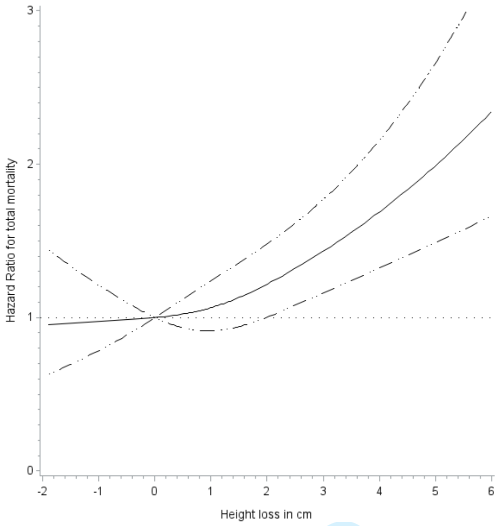

b) Total CVD mortality

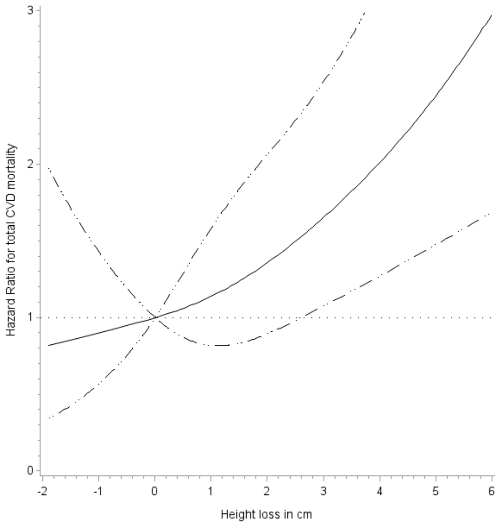

c) Stroke mortality

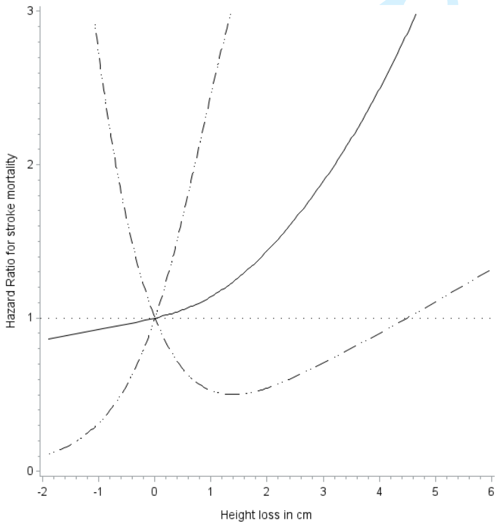

d) Non-CVD mortality

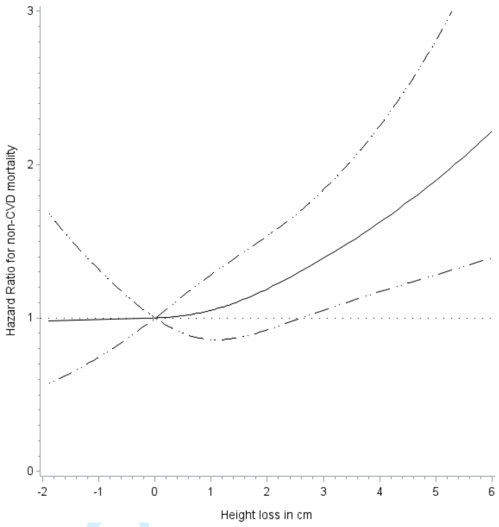

Supplemental table S1. Hazard ratios[a] and 95% confidence intervals for the association between height loss and total mortality in age-stratified samples of Swedish and Danish women (n=2406).

	**Total mortality**			
	**Model 1[b]**		**Model 2[c]**	
**Pooled sample stratified by baseline age[d]**	**< 50 years**	**≥ 50 years**	**< 50 years**	**≥ 50 years**
No. of cases /censored [a]	157/1183	468/598	157/1172	467/597
Height loss (cm)	1.14 (0.98, 1.34)	1.21 (1.14, 1.29)	1.14 (0.97; 1.34)	1.16 (1.08; 1.23)
Height loss (cm), binary:				
Stable (≤2)	1 (reference)	1 (reference)	1 (reference)	1 (reference)
Major height loss (>2)	1.90 (1.04, 3.46)	1.84 (1.48, 2.31)	2.02 (1.10; 3.68)	1.70 (1.36; 2.14)

[a] Number of deaths since second height measure / censored at end of follow-up. [b] Model 1 adjusted for age at follow-up, age^2, time interval and height at baseline. [c] Model 2 adjusted for age at follow-up, age^2, time interval, height at baseline, weight at baseline, baseline smoking, ethanol intake at baseline, baseline education and leisure time physical activity at both baseline and follow-up. [d] Significance of interaction term for continuous height loss*baseline age was ≥0.48 for both models. Significance of interaction term for binary height loss*baseline age was ≥0.61 for both models.

For peer review only

STROBE Statement—Checklist of items that should be included in reports of ***cohort studies***

	Item No	Recommendation	Reported on page #
**Title and abstract**	1	(*a*) Indicate the study's design with a commonly used term in the title or the abstract	1, 3
		(*b*) Provide in the abstract an informative and balanced summary of what was done and what was found	3-4
**Introduction**			
Background/rationale	2	Explain the scientific background and rationale for the investigation being reported	5
Objectives	3	State specific objectives, including any prespecified hypotheses	5
**Methods**			
Study design	4	Present key elements of study design early in the paper	6
Setting	5	Describe the setting, locations, and relevant dates, including periods of recruitment, exposure, follow-up, and data collection	6, 9
Participants	6	(*a*) Give the eligibility criteria, and the sources and methods of selection of participants. Describe methods of follow-up	6
		(*b*) For matched studies, give matching criteria and number of exposed and unexposed	Not applicable
Variables	7	Clearly define all outcomes, exposures, predictors, potential confounders, and effect modifiers. Give diagnostic criteria, if applicable	7-9
Data sources/ measurement	8*	For each variable of interest, give sources of data and details of methods of assessment (measurement). Describe comparability of assessment methods if there is more than one group	7-9
Bias	9	Describe any efforts to address potential sources of bias	9
Study size	10	Explain how the study size was arrived at	7
Quantitative variables	11	Explain how quantitative variables were handled in the analyses. If applicable, describe which groupings were chosen and why	7-9
Statistical methods	12	(*a*) Describe all statistical methods, including those used to control for confounding	9-10
		(*b*) Describe any methods used to examine subgroups and interactions	10
		(*c*) Explain how missing data were addressed	9
		(*d*) If applicable, explain how loss to follow-up was addressed	9
		(*e*) Describe any sensitivity analyses	10
**Results**			
Participants	13*	(a) Report numbers of individuals at each stage of study—eg numbers potentially eligible, examined for eligibility, confirmed eligible, included in the study, completing follow-up, and analysed	6
		(b) Give reasons for non-participation at each stage	6
		(c) Consider use of a flow diagram	-
Descriptive data	14*	(a) Give characteristics of study participants (eg demographic, clinical, social) and information on exposures and potential confounders	Table 1
		(b) Indicate number of participants with missing data for each variable of interest	Table 1, p. 9

For peer review only - http://bmjopen.bmj.com/site/about/guidelines.xhtml

		(c) Summarise follow-up time (eg, average and total amount)	p. 9, 11
Outcome data	15*	Report numbers of outcome events or summary measures over time	p. 11
Main results	16	(*a*) Give unadjusted estimates and, if applicable, confounder-adjusted estimates and their precision (eg, 95% confidence interval). Make clear which confounders were adjusted for and why they were included	Table 2, p. 7
		(*b*) Report category boundaries when continuous variables were categorized	p. 6, 9
		(*c*) If relevant, consider translating estimates of relative risk into absolute risk for a meaningful time period	-
Other analyses	17	Report other analyses done—eg analyses of subgroups and interactions, and sensitivity analyses	p. 11
**Discussion**			
Key results	18	Summarise key results with reference to study objectives	p. 12
Limitations	19	Discuss limitations of the study, taking into account sources of potential bias or imprecision. Discuss both direction and magnitude of any potential bias	p. 12
Interpretation	20	Give a cautious overall interpretation of results considering objectives, limitations, multiplicity of analyses, results from similar studies, and other relevant evidence	p. 13-16
Generalisability	21	Discuss the generalisability (external validity) of the study results	p. 15
**Other information**			
Funding	22	Give the source of funding and the role of the funders for the present study and, if applicable, for the original study on which the present article is based	

*Give information separately for exposed and unexposed groups.

Note: An Explanation and Elaboration article discusses each checklist item and gives methodological background and published examples of transparent reporting. The STROBE checklist is best used in conjunction with this article (freely available on the Web sites of PLoS Medicine at http://www.plosmedicine.org/, Annals of Internal Medicine at http://www.annals.org/, and Epidemiology at http://www.epidem.com/). Information on the STROBE Initiative is available at http://www.strobe-statement.org.

For peer review only

For peer review only - http://bmjopen.bmj.com/site/about/guidelines.xhtml

Loss of height in relation to total and cardiovascular mortality: a cohort study of northern European women

Journal:	*BMJ Open*
Manuscript ID	bmjopen-2021-049122.R2
Article Type:	Original research
Date Submitted by the Author:	26-Jun-2021

Complete List of Authors:	Klingberg, Sofia; University of Gothenburg, Department of Internal Medicine and Clinical Nutrition; University of Gothenburg, School of Public Health and Community Medicine<br>Mehlig, Kirsten; University of Gothenburg, School of Public Health and Community Medicine<br>Dangol, Rojina; Bispebjerg and Fredriksberg Hospitals, The Parker Institute, and the Center for Clinical Research and Prevention<br>Björkelund, Cecilia; University of Gothenburg Sahlgrenska Academy, Primary Health Care/School of Public Health and Community Medicine<br>Heitmann, Berit; Bispebjerg and Fredriksberg Hospitals, The Parker Institute, and the Center for Clinical Research and Prevention; University of Copenhagen Department of Public Health, Section for General Practice<br>Lissner, Lauren; University of Gothenburg, School of Public Health and Community Medicine

**Primary Subject Heading**:	Epidemiology
Secondary Subject Heading:	Cardiovascular medicine, Public health
Keywords:	EPIDEMIOLOGY, PREVENTIVE MEDICINE, PUBLIC HEALTH, CARDIOLOGY

I, the Submitting Author has the right to grant and does grant on behalf of all authors of the Work (as defined in the below author licence), an exclusive licence and/or a non-exclusive licence for contributions from authors who are: i) UK Crown employees; ii) where BMJ has agreed a CC-BY licence shall apply, and/or iii) in accordance with the terms applicable for US Federal Government officers or employees acting as part of their official duties; on a worldwide, perpetual, irrevocable, royalty-free basis to BMJ Publishing Group Ltd ("BMJ") its licensees and where the relevant Journal is co-owned by BMJ to the co-owners of the Journal, to publish the Work in this journal and any other BMJ products and to exploit all rights, as set out in our licence.

The Submitting Author accepts and understands that any supply made under these terms is made by BMJ to the Submitting Author unless you are acting as an employee on behalf of your employer or a postgraduate student of an affiliated institution which is paying any applicable article publishing charge ("APC") for Open Access articles. Where the Submitting Author wishes to make the Work available on an Open Access basis (and intends to pay the relevant APC), the terms of reuse of such Open Access shall be governed by a Creative Commons licence – details of these licences and which Creative Commons licence will apply to this Work are set out in our licence referred to above.

Other than as permitted in any relevant BMJ Author's Self Archiving Policies, I confirm this Work has not been accepted for publication elsewhere, is not being considered for publication elsewhere and does not duplicate material already published. I confirm all authors consent to publication of this Work and authorise the granting of this licence.

For peer review only

Loss of height in relation to total and cardiovascular mortality: a cohort study of northern European women

Sofia Klingberg[1, 2], Kirsten Mehlig[2], Rojina Dangol[3], Cecilia Björkelund[2], Berit L. Heitmann[3,4,5,*] and Lauren Lissner[2,*].

[1] Department of Internal Medicine and Clinical Nutrition, Institute of Medicine, Sahlgrenska Academy, University of Gothenburg, Gothenburg, Sweden

[2] School of Public Health and Community Medicine, Institute of Medicine, Sahlgrenska Academy, University of Gothenburg, Gothenburg, Sweden.

[3] The Parker Institute, and the Center for Clinical Research and Prevention, Bispebjerg and Fredriksberg Hospitals, The Capital Region, Denmark.

[4] Section for General Practice, Department of Public Health, University of Copenhagen, Denmark.

[5] The Boden Institute of Obesity, Nutrition, Exercise & Eating Disorders, Sydney Medical School, Sydney, Australia

*Shared last authorship

Corresponding author: Sofia Klingberg, Department of Internal Medicine and Clinical Nutrition, Institute of Medicine, Sahlgrenska Academy, University of Gothenburg, P.O Box 459, 405 30 Gothenburg, Sweden. sofia.klingberg@gu.se

Transparency statement: SK affirms that the manuscript is an honest, accurate, and transparent account of the study being reported and that no important aspects of the study have been omitted.

Keywords: height loss, longitudinal, women, mortality, cardiovascular disease

For peer review only - http://bmjopen.bmj.com/site/about/guidelines.xhtml

Abstract

Objective: To examine height changes in middle-aged northern European women in relation to overall and cause-specific mortality.

Design: Population-based cohort studies with longitudinally measured heights and register-based mortality.

Setting: Sweden and Denmark.

Participants: Population-based samples of 2406 Swedish and Danish women born on selected years 1908-1952, recruited to baseline examinations at ages 30-60, and re-examined 10-13 years later.

Main outcome measure: Total and CVD specific mortality during 17-19 years of follow-up after last height measure.

Results: For each 1 cm height loss during 10-13 years, the hazard ratio (HR) (95% CI) for total mortality was 1.14 (1.05 to 1.23) in Swedish women and 1.21 (1.09 to 1.35) in Danish women, independent of key covariates. Multivariable analyses revealed that low height and high leisure time physical activity at baseline were independently protective of height loss. Considering total mortality, the HR for major height loss, defined as height loss > 2 cm, were 1.74 (1.32 to 2.29) in Swedish women and 1.80 (1.27 to 2.54) in Danish women. Pooled analyses indicated that height loss was monotonically associated with an increased mortality, confirming a significant effect above 2 cm height loss. For cause-specific mortality, major height loss was associated with a HR of 2.31 (1.09 to 4.87) for stroke mortality, 2.14 (1.47 to 3.12) for total CVD mortality and 1.71 (1.28 to 2.29) for mortality due to causes other than CVD.

For peer review only

Conclusion: Height loss is a marker for excess mortality in northern European women. Specifically the hazard of CVD mortality is increased in women with height loss during middle age, and the results suggest that the strongest cause-specific endpoint may be stroke mortality. The present findings suggest attention to height loss in early- and mid-adulthood to identify women at high-risk of CVD, and that regular physical activity may prevent early onset height loss.

Strengths and limitations of the study

- The population-based sampling, the prospective designs, the standardized measurement protocols and the long durations of follow-up for endpoints through high-quality national registries are major strengths of this study.
- The number of deaths due to stroke was low and results regarding stroke should therefore be interpreted with some caution.
- Based on the observational design we cannot rule out residual confounding by unmeasured factors, such as early life exposure of physical activity and smoking, peak bone mass, diseases and medical treatments.

For peer review only - http://bmjopen.bmj.com/site/about/guidelines.xhtml

Introduction

Adult height is generally maintained from the end of puberty until the beginning of the 5th decade at which time height starts to decline (1-4). Height loss is a process caused by shrinking of vertebral discs (5), spinal compression fractures (6) and change in posture (7), accelerating from the 7th decade of life (1-4). Height loss is a predictor of both low bone mineral density (6) and clinical manifestations of osteoporotic fractures, which show a gradient across the northern-southern latitude (8).

Although height loss could be thought of as part of the normal ageing process, a rapid decline has been suggested to predict overall mortality risk in two studies of men (9, 10), one in both sexes combined (11) and a single study specifically in women (12). Height loss has also been specifically associated with deaths due to cardiovascular disease (CVD) in men (10) and sexes combined (11). However, most studies have been performed in older populations and no study has to our knowledge reported sex-specific estimates for women followed from early- to mid-adulthood. The fact that the effects of height loss in women has not been studied more thoroughly is remarkable since women tend to lose more height than men (1-4). Thus, the aim of the present study was to determine if height loss in mid-life predicts overall mortality as well as mortality due to CVD in two longitudinal, population-based samples of middle-aged Nordic women.

For peer review only - http://bmjopen.bmj.com/site/about/guidelines.xhtml

For peer review only

Methods

Study populations

Data from two prospective cohort studies were analyzed: the Swedish Prospective Population Study of Women in Gothenburg (PPSWG) and the female cohort of the Danish arm of the MONItoring trends and determinants of CArdiovascular disease (MONICA) study. The analyses were performed in each cohort separately and after pooling of the cohorts.

The PPSWG, initiated in 1968-1969, recruited a sample of 1462 women, born in 1908, 1914, 1918, 1922 and 1930 on specific dates that were distributed evenly across birth years. Over 90% of the invited women participated at the baseline health examination (13). In 1980-81, a re-examination took place. A total of 1153 women were examined in PPSWG at both time-points, and were thus eligible for the present study.

The Danish MONICA study recruited 1765 randomly selected female residents of western Copenhagen born in 1922, 1932, 1942 and 1952 to a health examination in 1982-1984 (14). The baseline participation rate was 79% (14). A re-examination took place in 1994, in which 1264 women took part, constituting the eligible sample from MONICA.

The total number of Swedish and Danish women for this study was thus 2417. Exclusions of six Swedish and five Danish women due to implausible height increase of ≥ 2 cm, left a final analytical sample of 2406 women.

For peer review only - http://bmjopen.bmj.com/site/about/guidelines.xhtml

Height and other covariates

At both examinations, height was measured with a stadiometer to the nearest 0.5 cm on subjects without shoes. Measurement of height was generally performed early in the day. Height loss was calculated as baseline height minus height at follow-up. Thus, a positive value indicated height loss.

Potential confounders (age, time between measures, and baseline measures of height, weight, smoking, leisure time physical activity (LTPA), ethanol intake and education) were selected based on their known associations with height loss and mortality. All confounders were fully harmonized between the studies and included in fully adjusted models. Time between measures was calculated as the difference in time between follow-up measure and baseline measure. Participants were weighed wearing light clothing on a calibrated scale. Information on all other covariates was collected by questionnaires. Smoking status was categorized into never, former, and current smokers. LTPA was assessed by a question placing the participants in one of four categories ranging from sedentary to vigorous activity (15). Participants were then classified into three categories: almost inactive (low LTPA); at least four hours of low impact physical activity per week (medium LTPA); or regular physical exercise or competitive sports (high LTPA). Habitual intake of beer, wine and spirits during the last year was assessed based on which ethanol intake per day was estimated. Educational level was dichotomized into compulsory education vs. more than compulsory. In PPSWG compulsory education ranged from 6 to 7 years in the different birth cohorts,

For peer review only - http://bmjopen.bmj.com/site/about/guidelines.xhtml

while 7 years of schooling was compulsory for all birth cohorts in MONICA. Information on menopausal status was available but not included because of the collinearity with age.

End points

Date and cause of death were assessed through national mortality registries. The Danish cohort was followed for total mortality until Oct 4th 2012 with a maximum follow-up of 19.3 years, and until 31st Dec 2010 for cause specific mortality with a maximum follow-up of 16.7 years. The Swedish cohort was followed until 2014, but follow-up was restricted to 19.3 and 16.7 years for total mortality and cause specific mortality, respectively, to harmonize with the Danish cohort. Death due to CVD was identified by ICD-8/9 codes 390 to 459 or ICD-10 codes I00-I99, and death specifically due to stroke was identified by ICD-8/9 codes 430 to 434 or ICD-10 codes I60-I64. Unspecified or uncertain stroke diagnoses in the Swedish cohort were verified through medical records (16).

For peer review only

Statistical analyses

The Cox proportional hazards model was used to investigate if height loss was associated with overall mortality as well as cause-specific mortality. Time of follow-up from last height measure until date of death or censoring was used as underlying time metric. Height loss was parameterized both as linear predictor and as binary predictor. Height loss was dichotomized at 2 cm, defining major height loss by height loss >2 cm. Restricted cubic spline regression was used to further investigate non-linear associations between height loss and hazard ratio (HR) for the outcomes. Three knots were automatically placed at: -0.7; 0.8; and 2.5 cm, with the reference set to zero (no height loss). Because of missing data for smoking, LTPA, education and/or ethanol intake 74 Danish and 12 Swedish women were excluded from models that adjusted for these covariates. The proportional hazards assumption (PHA) of the Cox model was tested by inclusion of a product term between the major height loss variable and survival time until death or censoring for the respective outcome. These tests did not indicate violation of the PHA (all p>0.64). Linear regression and logistic regression were used for investigation of association between covariates and height loss.

For peer review only

In order to explore effect modification by baseline age, interactions between age (dichotomized at <50 vs. ≥50 years) and height loss (both linear and dichotomized) on overall mortality were investigated. To rule out influence of large height losses on the linear analyses, women with height loss ≥ 5 cm and ≥ 4 cm, respectively, were excluded in sensitivity analyses of the main outcome, total mortality. Analyses were also repeated after exclusion of deaths within the first two years after start of follow up.

For peer review only - http://bmjopen.bmj.com/site/about/guidelines.xhtml

Interactions between cohort and height loss on total and cause-specific mortality were examined by inclusion of the corresponding product term in models of both linear and dichotomized height loss.

All statistical analyses were performed in SAS, version 9.3 (SAS Institute, Cary, NC). Results with p-values of <0.05 was considered significant (two-sided test). A HR was considered significant when the 95% confidence interval (CI) did not include 1.

Patient and public involvement

Patients or the public were not involved in this specific research project.

For peer review only

Results

Characteristics of the study population are presented in Table 1. At baseline, the average ages of the Swedish and Danish cohorts were 47 and 44 years, respectively and two-thirds were aged 38-52 years at baseline. In the pooled cohort, women lost on average 0.8 cm of height (range -1.9 to 14.0) over 11.4 years. During follow-up over a maximum of 19.3 years, 316 and 309 cases of all-cause mortality occurred in the Swedish and Danish cohorts, respectively. Considering cause-specific mortality over a maximum of 16.7 years, CVD was the primary cause in 157 cases, of which 37 were specifically due to stroke, while 362 cases were due to non-CVD causes (in total 519 deaths).

The first stage of the analyses examined mortality irrespective of cause. The hazard for total mortality for each cm of height loss was 1.18 (1.09 to 1.28) for the Swedish women and 1.24 (1.13 to 1.37) for the Danish women, after adjusting for age, baseline height and time between height measures (Table 2). Further adjustment for baseline weight and lifestyle factors gave similar estimates. Major height loss, defined as height loss > 2cm, was associated with 74 and 80% higher hazard for total mortality in Swedish and Danish women, respectively. Based on the similarities of the separate results, we pooled the cohorts. Pooled analyses suggested that height loss was associated with a monotonic increase in total mortality hazard (Figure 1) with a significant effect of a height loss of > 2 cm. Inclusion of interaction terms between cohort and height loss in pooled analyses showed no sign of spatial heterogeneity (p-values for interaction >0.50 for total mortality). Age (year) as linear factor was associated with total mortality in pooled analyses (HR (95% CI) 1.12 (1.11 to 1.14)) (data not shown). Stratification by baseline age showed that the effect was similar in

For peer review only - http://bmjopen.bmj.com/site/about/guidelines.xhtml

For peer review only

women aged <50 and ≥50 years at baseline in models with continuous height (interactions p≥0.48) as well as dichotomized height (interactions p≥0.61) (Table S1).

All further analyses on cause-specific mortality were performed on the pooled sample due to the limited number of outcomes. These analyses suggested that continuous height loss was associated with total CVD mortality, stroke mortality and non-CVD mortality (Table 2). Major height loss was associated with a HR (95% CI) of 2.31 (1.09 to 4.87) for stroke mortality, 2.14 (1.47 to 3.12) for total CVD mortality and 1.71 (1.28 to 2.29) for mortality due to other causes, independent of age, time interval between height measures, baseline height, baseline weight, cohort and lifestyle factors (Table 2). Stroke mortality did, however, not account for the effect of height loss on overall mortality, since height loss also was associated with non-stroke CVD mortality (HR (95% CI) 1.82 (1.43 to 2.32) (data not shown). No significant interactions were found between height loss and cohort on cause-specific mortality (p-values for interaction >0.20 for all cause-specific mortality outcomes).

Sensitivity analyses were performed to rule out the possibility that the continuous effect of decreased height on overall mortality was driven by large decreases in only few individuals. Excluding women with a loss of height ≥5 cm (n=11) did not weaken the effect estimate of linear height on total mortality HR (95% CI) 1.21 (1.10 to 1.34). Similarly, when further excluding 14 women with a loss of ≥4 cm, the effect estimate remained at the same magnitude 1.19 (1.08 to 1.32). Excluding deaths occurring during the first two years of follow up (21 cases of total mortality, 6 cases of CVD mortality and 15 cases of non-CVD mortality) did not alter the results (data not shown).

For peer review only - http://bmjopen.bmj.com/site/about/guidelines.xhtml

For peer review only

Age-adjusted associations between covariates and height loss were also investigated (data not shown). Baseline height was consistently associated to greater subsequent height loss, both linear height loss (β=0.007, p=0.03) and major height loss (OR (95% CI) 1.03 (1.00 to 1.06). Aside from baseline height, the only other factor associated to height loss was baseline LTPA. High baseline LTPA was, compared to medium baseline LTPA, linearly associated with lesser height loss (β=-0.15, p=0.02) while the association with major height loss was not significant (OR (95% CI) 0.85 (0.52 to 1.37). In contrast, low baseline LTPA was, compared to medium baseline LTPA, not associated with height loss (data not further shown).

Discussion

Statement of principal findings

In this study of middle-aged Nordic women, major height loss was associated with an elevated hazard of overall mortality of around 80%. Specifically, major height loss associated with CVD mortality, with more than a 2-fold risk for stroke mortality. The findings were independent of age, time between height measures, cohort, and baseline values of height, weight, education and lifestyle factors.

Strengths and limitations of the study

The main strengths of this study are its population-based sampling, the prospective designs, the standardized measurement protocols and the long durations of follow-up for endpoints. Furthermore, trained staff measured height using standardized methodology, and mortality was ascertained through high-quality national registries (17, 18), thereby limiting potential

For peer review only - http://bmjopen.bmj.com/site/about/guidelines.xhtml

bias in both exposure and outcome. Also, differences between the cohorts in study design, age and period were minor with results for overall mortality that were strikingly similar in both samples, and results for CVD mortality that were independent of time between height measures, age at follow-up and cohort. However, we acknowledge the risk for bias due to non-participation in both cohorts. We performed both stratified and pooled analyses, and investigated interactions by cohort, with consistent results in both cohorts. The consistency of cohort-specific results may indicate that non-participation has a minor influence on the association analyses. Another limitation is that deaths due to stroke were quite few, which implies that these results should be interpreted with some caution. Additionally, based on the observational design we cannot rule out residual confounding by unmeasured factors, such as early life exposure of physical activity and smoking, peak bone mass, diseases and medical treatments. Based on the fact that bone health is different depending on ethnicity it is important to take this factor into account when interpreting the results and generalizing the results. In Monica, subjects with non-Danish origin were not included (14), and in the Swedish cohort only 0.3 % were born outside Europe (13). Hence, it can be concluded that the vast majority of participants were Caucasians.

Comparison with other studies and interpretation

To the best of our knowledge, this study is the first to report results on the effect of height loss on mortality in women followed from middle age. Previous studies of men and mixed samples, including populations followed from a baseline age of 40 years, have shown height loss to associate with total mortality with risk ratios or HR between 1.45 and 3.43 (9-11). Previous studies of female populations were started at older age and their results are discordant. Hillier et al showed that in a cohort of women aged ≥65 years at baseline, losing

For peer review only - http://bmjopen.bmj.com/site/about/guidelines.xhtml

≥ 5 cm over 15 years was associated with an increased hazard for mortality of 45%, compared to women losing less (12). However, another study of women of the same baseline age found no significant effect of 4-year height loss of > 2 cm (9). The lack of association in that study could possibly be explained by low power due to few women classified as exposed and additionally the low number of deaths during follow-up. Compared to the above-mentioned studies, the present study shows overall mortality estimates within the range of those previously reported, indicating a consistent association between height loss and mortality in both women and men and over the adult life course. The latter was further confirmed by the congruent results in women <50 years and ≥ 50 years in the present study.

Moreover, the present study points out that height loss specifically predicts CVD mortality, whereas results from previous studies are somewhat divergent. Wannamethee et al found an increased risk of CVD mortality in men (10). This was confirmed by Masunari et al who presented results for women and men together (11). On the contrary, Auyeng et al found no association in neither men nor women (9), but yet again, the number of exposed as well as cases in this study was low. To our knowledge, the novel finding in the present study showing a particular association between height loss and stroke mortality has not been reported before. Wannamethee et al investigated height loss in relation to incident major stroke events in men, but found no such association (10). Furthermore, height loss and CVD are linked, both epidemiologically and mechanistically, by interrelations between bone loss or osteoporosis and CVD. A recent systematic review and meta-analysis, found that low baseline bone mineral density (BMD), and fractures, were associated to an increased risk of

For peer review only - http://bmjopen.bmj.com/site/about/guidelines.xhtml

developing CVD (19). The pathophysiological links between these conditions are not fully understood but presumably involve chronic inflammation and oxidative stress (20). Additionally, similarities exist in the process of bone formation and vascular calcification, including involvement of a range of bone biomarkers (20). Frailty, a clinical syndrome defined by impaired physical resources (21), is another feature linked to both osteoporosis (22) and CVD (23). Different definitions of frailty exist but one of the most commonly used is the one operationalized by Fried et al. in which frailty is defined by the occurrence of three out of five criteria: unintentional weight loss, self-reported exhaustion, weakness, slow walking speed, and low physical activity (21), but not height loss. Weakness has been shown as a predominant initial sign of frailty (24). Weakness could be attributed to sarcopenia, a muscle disease common in older adults diagnosed by low muscle function in the presence of low muscle quantity or quality (25). Sarcopenia is an age-related process, but can also stem from inflammation, malnutrition, and physical inactivity (25). Thus, height loss is not recognized in the definition of frailty but it should be highlighted that height loss shares both the feature of impaired physical resources and the etiology for sarcopenia and therefore future frailty definitions could consider height loss as a potential important criteria.

Implications of the findings

The results from the present study may be generalized to northern latitude Caucasian women. Within the northern latitude, British men have previously been investigated (10). Populations within the northern latitude are over-represented when it comes to osteoporotic fractures (8) and, although we have not been able to find publications on

For peer review only - http://bmjopen.bmj.com/site/about/guidelines.xhtml

For peer review only

differences of height loss across latitudes, it could be hypothesized that populations within the northern latitude lose more height when aging. To gain further understanding, more studies of women as well as men from this region are warranted to improve knowledge about the relation between height loss, morbidity and mortality.

Height is a simple measure that could be taken in every clinical setting, as compared to for example measurement of BMD, which acquires advanced methodology. Despite its simplicity, height measurement is rarely included in the clinical examination at the general practitioner. Taken together, these results suggest that height loss should be recognized within primary care to facilitate actions for CVD prevention, but others also indicate height loss as an important indicator of low BMD, vertebral fractures and vitamin D deficiency (6).

Knowledge on how to prevent height loss is sparse. Pharmaceutical treatment for osteoporosis with alendronate has shown to prevent height loss in addition to improving bone mineral content (26), while supplement with calcium and vitamin D has not proven to prevent height loss (27). Concerning lifestyle, physical activity has been identified as protective against height loss in post-menopausal women (28). Our results confirmed that regular physical exercise could contribute significantly to height loss prevention. Still, these results suggest that moderate activity may not be enough to prevent height loss and only one in seven women in the current cohorts were active enough to benefit from physical activity in relation to decreased height loss. More research is thus needed, not only on the consequences of height loss but also on the causes in order to facilitate prevention of height loss and associated comorbidity and mortality.

For peer review only - http://bmjopen.bmj.com/site/about/guidelines.xhtml

Conclusions

Height loss during mid-life is a risk marker for earlier mortality in northern European women. Specifically the hazard of CVD mortality is increased in women with height loss, and the results suggested that stroke mortality may be a major contributor to the total CVD association. These findings suggest the need for increased attention to height loss to identify individuals at increased CVD risk. Moreover, regular physical activity may be beneficial not only in prevention of CVD, but also in prevention of height loss and thereby further contributing to CVD prevention.

Ethical approval statement: Participants from both study cohorts provided informed consent to participate. Since 1980, all examinations in PPSWG have been approved by the Regional Ethics Review Board in Gothenburg, in accordance with the Declaration of Helsinki (registration number T331-14 for linkage to national registries). The MONICA study was approved by the Local Ethics Committee of Copenhagen County and the Danish Data protection Office (J.nr 2015-41-3942), and was in accordance with the principles of the Helsinki Declaration.

Contributorship statement: BLH and LL initiated the project. SK and KM performed all statistical analyses. SK had main responsibility for writing the article. SK, KM, RD, CB, BLH and LL all contributed to the statistical analyses and interpretation and provided comments on the manuscript. SK, KM, RD, CB, BLH and LL all read and approved the final manuscript.

Competing interest: The authors declare no conflict of interest.

For peer review only - http://bmjopen.bmj.com/site/about/guidelines.xhtml

Funding statement: This work was supported by grants from the Swedish Research Council (VR521-2010-2984), the Swedish Research Council for Health, working Life, and Welfare (2006-1506), and the Swedish ALF-agreement (ALFGBG-672971 and ALFGBG-722441). The funding sources had no role in study design, collection, analysis and interpretation of data, the writing of the report, or the decision to submit the article for publication. The authors declare no conflict of interest.

Data availability statement: Data underlying this article are available upon reasonable request. Data cannot be made publicly available for ethical and legal reasons. Public availability may compromise participant privacy, and this would not comply with Danish or Swedish legislation. Requests for data should be addressed to Professor Berit L Heitmann (Berit.Lilienthal.Heitmann@regionh.dk) and Professor Lauren Lissner (lauren.lissner@gu.se) who will provide the data access in accordance with the Danish and Swedish Data Protection Agency, respectively.

For peer review only

References

1. Cline MG, Meredith KE, Boyer JT, Burrows B. Decline of height with age in adults in a general population sample: estimating maximum height and distinguishing birth cohort effects from actual loss of stature with aging. Hum Biol. 1989;61(3):415-25.
2. Sorkin JD, Muller DC, Andres R. Longitudinal change in height of men and women: implications for interpretation of the body mass index: the Baltimore Longitudinal Study of Aging. American journal of epidemiology. 1999;150(9):969-77.
3. Peter RS, Fromm E, Klenk J, Concin H, Nagel G. Change in height, weight, and body mass index: longitudinal data from Austria. Am J Hum Biol. 2014;26(5):690-6.
4. Droyvold WB, Nilsen TI, Kruger O, Holmen TL, Krokstad S, Midthjell K, et al. Change in height, weight and body mass index: Longitudinal data from the HUNT Study in Norway. Int J Obes (Lond). 2006;30(6):935-9.
5. Pfirrmann CW, Metzdorf A, Elfering A, Hodler J, Boos N. Effect of aging and degeneration on disc volume and shape: A quantitative study in asymptomatic volunteers. Journal of orthopaedic research : official publication of the Orthopaedic Research Society. 2006;24(5):1086-94.
6. Mikula AL, Hetzel SJ, Binkley N, Anderson PA. Validity of height loss as a predictor for prevalent vertebral fractures, low bone mineral density, and vitamin D deficiency. Osteoporosis international : a journal established as result of cooperation between the European Foundation for Osteoporosis and the National Osteoporosis Foundation of the USA. 2017;28(5):1659-65.
7. Tsunoda K, Research Group on the Relationship of Bent P, Stroke NHO. Height loss caused by bent posture: a risk factor for stroke from ENT clinic - is it time to reconsider the physical examination? Acta Otolaryngol. 2011;131(10):1079-85.
8. Kanis JA, Oden A, McCloskey EV, Johansson H, Wahl DA, Cooper C, et al. A systematic review of hip fracture incidence and probability of fracture worldwide. Osteoporosis international : a journal established as result of cooperation between the European Foundation for Osteoporosis and the National Osteoporosis Foundation of the USA. 2012;23(9):2239-56.
9. Auyeung TW, Lee JS, Leung J, Kwok T, Leung PC, Woo J. Effects of height loss on morbidity and mortality in 3145 community-dwelling Chinese older women and men: a 5-year prospective study. Age Ageing. 2010;39(6):699-704.
10. Wannamethee SG, Shaper AG, Lennon L, Whincup PH. Height loss in older men: associations with total mortality and incidence of cardiovascular disease. Archives of internal medicine. 2006;166(22):2546-52.
11. Masunari N, Fujiwara S, Kasagi F, Takahashi I, Yamada M, Nakamura T. Height loss starting in middle age predicts increased mortality in the elderly. Journal of bone and mineral research : the official journal of the American Society for Bone and Mineral Research. 2012;27(1):138-45.
12. Hillier TA, Lui LY, Kado DM, LeBlanc ES, Vesco KK, Bauer DC, et al. Height loss in older women: risk of hip fracture and mortality independent of vertebral fractures. Journal of bone and mineral research : the official journal of the American Society for Bone and Mineral Research. 2012;27(1):153-9.
13. Bengtsson C, Blohme G, Hallberg L, Hällström T, Isaksson B, Korsan-Bengtsen K, et al. The study of women in Gothenburg 1968-1969--a population study. General design, purpose and sampling results. Acta medica Scandinavica. 1973;193(4):311-8.
14. Jorgensen T. Prevalence of gallstones in a Danish population. American journal of epidemiology. 1987;126(5):912-21.
15. Grimby G, Börjesson M, Jonsdottir IH, Schnohr P, Thelle DS, Saltin B. The "Saltin-Grimby Physical Activity Level Scale" and its application to health research. Scandinavian journal of medicine & science in sports. 2015;25 Suppl 4:119-25.

For peer review only - http://bmjopen.bmj.com/site/about/guidelines.xhtml

16. Blomstrand A, Blomstrand C, Ariai N, Bengtsson C, Björkelund C. Stroke incidence and association with risk factors in women: a 32-year follow-up of the Prospective Population Study of Women in Gothenburg. BMJ Open. 2014;4(10):e005173.
17. Brooke HL, Talback M, Hornblad J, Johansson LA, Ludvigsson JF, Druid H, et al. The Swedish cause of death register. European journal of epidemiology. 2017;32(9):765-73.
18. Helweg-Larsen K. The Danish Register of Causes of Death. Scand J Public Health. 2011;39(7 Suppl):26-9.
19. Veronese N, Stubbs B, Crepaldi G, Solmi M, Cooper C, Harvey NC, et al. Relationship Between Low Bone Mineral Density and Fractures With Incident Cardiovascular Disease: A Systematic Review and Meta-Analysis. Journal of bone and mineral research : the official journal of the American Society for Bone and Mineral Research. 2017;32(5):1126-35.
20. Vassalle C, Mazzone A. Bone loss and vascular calcification: A bi-directional interplay? Vascul Pharmacol. 2016;86:77-86.
21. Fried LP, Tangen CM, Walston J, Newman AB, Hirsch C, Gottdiener J, et al. Frailty in older adults: evidence for a phenotype. The journals of gerontology Series A, Biological sciences and medical sciences. 2001;56(3):M146-56.
22. Li G, Thabane L, Papaioannou A, Ioannidis G, Levine MA, Adachi JD. An overview of osteoporosis and frailty in the elderly. BMC Musculoskelet Disord. 2017;18(1):46.
23. Stewart R. Cardiovascular Disease and Frailty: What Are the Mechanistic Links? Clinical chemistry. 2019;65(1):80-6.
24. Xue QL, Bandeen-Roche K, Varadhan R, Zhou J, Fried LP. Initial manifestations of frailty criteria and the development of frailty phenotype in the Women's Health and Aging Study II. The journals of gerontology Series A, Biological sciences and medical sciences. 2008;63(9):984-90.
25. Cruz-Jentoft AJ, Bahat G, Bauer J, Boirie Y, Bruyere O, Cederholm T, et al. Sarcopenia: revised European consensus on definition and diagnosis. Age Ageing. 2019;48(1):16-31.
26. Prinsloo PJ, Hosking DJ. Alendronate sodium in the management of osteoporosis. Ther Clin Risk Manag. 2006;2(3):235-49.
27. Crandall CJ, Aragaki AK, LeBoff MS, Li W, Wactawski-Wende J, Cauley JA, et al. Calcium plus vitamin D supplementation and height loss: findings from the Women's Health Initiative Calcium and Vitamin D clinical trial. Menopause. 2016;23(12):1277-86.
28. Mai X, Marshall B, Hovey KM, Sperrazza J, Wactawski-Wende J. Risk factors for 5-year prospective height loss among postmenopausal women. Menopause. 2018;25(8):883-9.

For peer review only

For peer review only - http://bmjopen.bmj.com/site/about/guidelines.xhtml

Table 1. Characteristics of the two study populations and the pooled sample.

		Swedish women (n=1147)	Danish women (n=1259)	Pooled sample (n=2406)
Age distribution at baseline (n (%))				
	30-32 years	-	358 (28.4)	358 (14.9)
	38-42 years	305 (26.6)	344 (27.3)	649 (27.0)
	46-47 years	332 (29.0)	-	332 (13.8)
	50-52 years	323 (28.2)	321 (25.5)	644 (26.8)
	54-55 years	138 (12.0)	-	138 (5.7)
	60-62 years	49 (4.3)	236 (18.8)	285 (11.9)
Age at baseline height examination (mean (sd), years)		47.1 (6.1)	44.3 (10.8)	45.6 (9.0)
Age at last height examination (mean (sd), years)		59.1 (6.1)	55.2 (10.8)	57.1 (9.1)
Height baseline (mean (sd), cm)		163.7 (5.8)	163.7 (6.1)	163.7 (6.0)
Weight baseline (mean (sd), kg)		64.3 (10.3)	63.1(10.5)	63.7 (10.4)
Height loss (mean (sd), cm)		1.09 (1.21)	0.6 (1.0)	0.84 (1.12)
Time interval (mean (sd), years)		12.0 (0.2)	10.9 (0.3)	11.4 (0.6)
Rate of height loss (mean (sd), cm/year)		0.09 (0.10)	0.06 (0.09)	0.07 (0.10)
Education: compulsory or less at baseline (N (%))		793 (69.3)	411 (32.6)	1204 (50.1)
Smoking at baseline (N (%))				
	Never	603 (52.6)	415 (33.0)	1018 (42.3)
	Former	89 (7.8)	215 (17.1)	304 (12.6)
	Current	454 (39.6)	629 (50.0)	1083 (45.0)
Leisure time physical activity at baseline (N (%))				
	Low (almost inactive)	198 (17.3)	367 (29.2)	565 (23.5)
	Medium (≥4h low impact leisure time physical activity/ week)	812 (71.0)	718 (57.3)	1530 (63.8)
	High (regular physical activity or competitive sports)	136 (11.9)	174 (13.8)	310 (12.9)
Leisure time physical activity at follow up (n (%))				
	Low (almost inactive)	335 (29.2)	311 (26.1)	646 (27.6)

For peer review only - http://bmjopen.bmj.com/site/about/guidelines.xhtml

For peer review only

	Medium (≥4h low impact leisure time physical activity/ week)	582 (51.0)	735 (61.8)	1317 (56.5)
	High (regular physical activity or competitive sports)	230 (20.1)	146 (12.3)	376 (16.1)
Ethanol intake (mean (sd), g/day)		8.3 (10.9)	9.8 (11.6)	9.1 (11.3)

Table 2. Hazard ratios[a] and 95% confidence intervals for the association between height loss and total mortality and cause-specific mortality in Swedish and Danish women (n=2406).

	Total mortality					
	**Model 1[b]**			**Model 2[c]**		
**Sample**	**Swedish**	**Danish**	**Pooled**	**Swedish**	**Danish**	**Pooled**
No. of cases /censored [a]	316/831	309/950	625/1781	314/821	292/893	606/1714
Height loss (cm)	1.18 (1.09, 1.28)	1.24 (1.13, 1.37)	1.20 (1.13, 1.27)	1.14 (1.05; 1.23)	1.21 (1.09; 1.35)	1.15 (1.09; 1.23)
Height loss (cm), binary:						
Stable (≤2)	1 (reference)	1 (reference)	1 (reference)	1 (reference)	1 (reference)	1 (reference)
Major height loss (>2)	1.86 (1.41, 2.44)	1.90 (1.36, 2.64)	1.85 (1.50, 2.29)	1.74 (1.32; 2.29)	1.80 (1.27; 2.54)	1.74 (1.41; 2.16)
	**Total CVD mortality**		**Stroke mortality**		**Non-CVD mortality**	
**Pooled sample**	**Model 1[b]**	**Model 2[c]**	**Model 1[b]**	**Model 2[c]**	**Model 1[b]**	**Model 2[c]**
No. of cases /censored [a]	157/2249	156/2164	37/2369	36/2284	362/2044	347/1973
Height loss (cm)	1.28 (1.17, 1.40)	1.21 (1.10; 1.32)	1.36 (1.15, 1.61)	1.30 (1.09; 1.55)	1.18 (1.09, 1.28)	1.14 (1.05; 1.24)
Height loss (cm), binary:						
Stable (≤2)	1 (reference)	1 (reference)	1 (reference)	1 (reference)	1 (reference)	1 (reference)
Major height loss (>2)	2.44 (1.69, 3.53)	2.14 (1.47; 3.12)	2.81 (1.38, 5.75)	2.31 (1.09; 4.87)	1.78 (1.34, 2.36)	1.71 (1.28; 2.29)

[a] Number of deaths since second height measure / censored at end of follow-up. [b] Model 1 adjusted for age at follow-up, age^2, time interval and height at baseline. Analyses of pooled sample also adjusted for cohort (Swedish/Danish). [c]Model 2 adjusted for age at follow-up, age^2, time interval, height at baseline, weight at baseline, baseline smoking, ethanol intake at baseline, baseline education and leisure time physical activity at both baseline and follow-up. Analyses of pooled sample also adjusted for cohort (Swedish/Danish).

For peer review only - http://bmjopen.bmj.com/site/about/guidelines.xhtml

Figure legend

Figure 1. Hazard ratio (HR) for 10-12 year decrease in height in relation to total mortality and cause-specific mortality in a pooled sample of Swedish and Danish women. Models were adjusted for age at follow-up, age^2, time interval between height measures, and height at baseline, weight at baseline, baseline smoking, ethanol intake at baseline, baseline education and leisure time physical activity at both baseline and follow-up. Zero change in height was used as reference value for HR. Dark line represents HR. Dotted lines represent 95% CI of HR. a) Total mortality. Test for curvature p=0.28, test for overall significance of curve p<0.001, test for linearity p<0.001. b) CVD mortality. Test for curvature p=0.72, test for overall significance of curve p<0.01, test for linearity p<0.001. c) Stroke mortality. Test for curvature p=0.74, test for overall significance of curve p=0.06, test for linearity p<0.05. d) Non-CVD mortality. Test for curvature p=0.39, test for overall significance of curve p<0.05, test for linearity p<0.01.

For peer review only

a) Total mortality

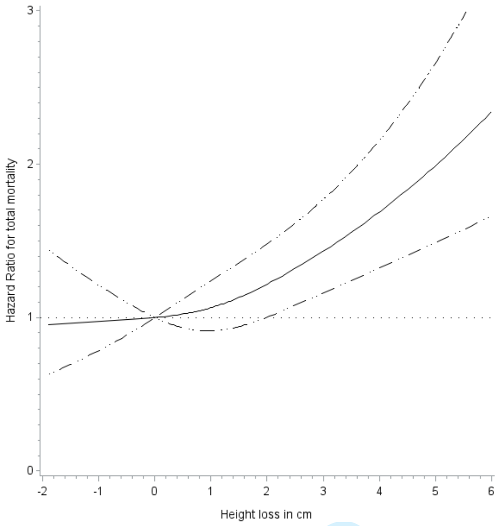

b) Total CVD mortality

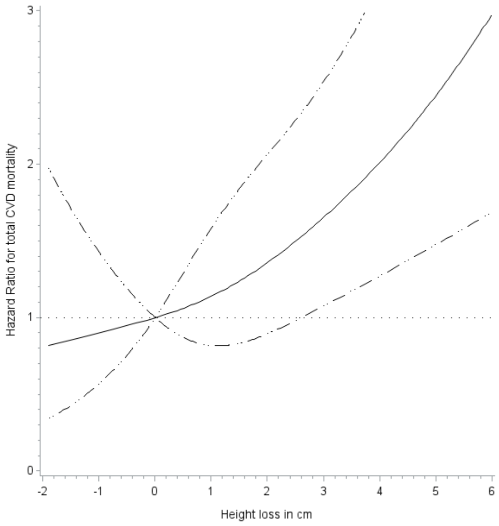

c) Stroke mortality

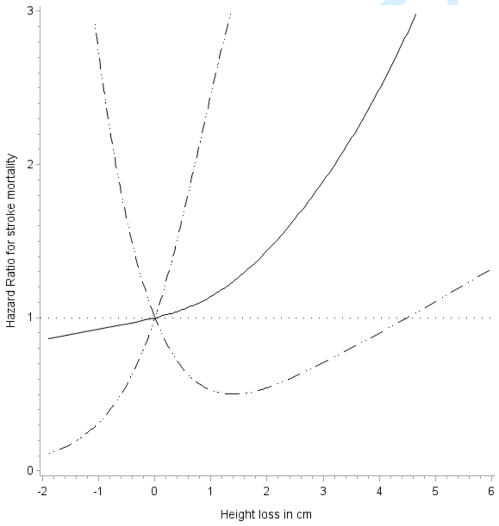

d) Non-CVD mortality

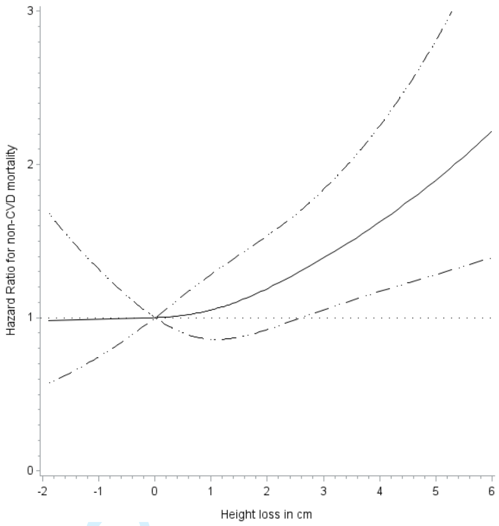

Supplemental table S1. Hazard ratios[a] and 95% confidence intervals for the association between height loss and total mortality in age-stratified samples of Swedish and Danish women (n=2406).

	**Total mortality**			
	**Model 1[b]**		**Model 2[c]**	
**Pooled sample stratified by baseline age[d]**	**< 50 years**	**≥ 50 years**	**< 50 years**	**≥ 50 years**
No. of cases /censored [a]	157/1183	468/598	157/1172	467/597
Height loss (cm)	1.14 (0.98, 1.34)	1.21 (1.14, 1.29)	1.14 (0.97; 1.34)	1.16 (1.08; 1.23)
Height loss (cm), binary:				
Stable (≤2)	1 (reference)	1 (reference)	1 (reference)	1 (reference)
Major height loss (>2)	1.90 (1.04, 3.46)	1.84 (1.48, 2.31)	2.02 (1.10; 3.68)	1.70 (1.36; 2.14)

[a] Number of deaths since second height measure / censored at end of follow-up. [b] Model 1 adjusted for age at follow-up, age^2, time interval and height at baseline. [c]Model 2 adjusted for age at follow-up, age^2, time interval, height at baseline, weight at baseline, baseline smoking, ethanol intake at baseline, baseline education and leisure time physical activity at both baseline and follow-up. [d] Significance of interaction term for continuous height loss*baseline age was ≥0.48 for both models. Significance of interaction term for binary height loss*baseline age was ≥0.61 for both models.

For peer review only

STROBE Statement—Checklist of items that should be included in reports of ***cohort studies***

	Item No	Recommendation	Reported on page #
**Title and abstract**	1	(*a*) Indicate the study's design with a commonly used term in the title or the abstract	1, 3
		(*b*) Provide in the abstract an informative and balanced summary of what was done and what was found	3-4
**Introduction**			
Background/rationale	2	Explain the scientific background and rationale for the investigation being reported	5
Objectives	3	State specific objectives, including any prespecified hypotheses	5
**Methods**			
Study design	4	Present key elements of study design early in the paper	6
Setting	5	Describe the setting, locations, and relevant dates, including periods of recruitment, exposure, follow-up, and data collection	6, 9
Participants	6	(*a*) Give the eligibility criteria, and the sources and methods of selection of participants. Describe methods of follow-up	6
		(*b*) For matched studies, give matching criteria and number of exposed and unexposed	Not applicable
Variables	7	Clearly define all outcomes, exposures, predictors, potential confounders, and effect modifiers. Give diagnostic criteria, if applicable	7-9
Data sources/ measurement	8*	For each variable of interest, give sources of data and details of methods of assessment (measurement). Describe comparability of assessment methods if there is more than one group	7-9
Bias	9	Describe any efforts to address potential sources of bias	9
Study size	10	Explain how the study size was arrived at	7
Quantitative variables	11	Explain how quantitative variables were handled in the analyses. If applicable, describe which groupings were chosen and why	7-9
Statistical methods	12	(*a*) Describe all statistical methods, including those used to control for confounding	9-10
		(*b*) Describe any methods used to examine subgroups and interactions	10
		(*c*) Explain how missing data were addressed	9
		(*d*) If applicable, explain how loss to follow-up was addressed	9
		(*e*) Describe any sensitivity analyses	10
**Results**			
Participants	13*	(a) Report numbers of individuals at each stage of study—eg numbers potentially eligible, examined for eligibility, confirmed eligible, included in the study, completing follow-up, and analysed	6
		(b) Give reasons for non-participation at each stage	6
		(c) Consider use of a flow diagram	-
Descriptive data	14*	(a) Give characteristics of study participants (eg demographic, clinical, social) and information on exposures and potential confounders	Table 1
		(b) Indicate number of participants with missing data for each variable of interest	Table 1, p. 9

For peer review only

For peer review only - http://bmjopen.bmj.com/site/about/guidelines.xhtml

		(c) Summarise follow-up time (eg, average and total amount)	p. 9, 11
Outcome data	15*	Report numbers of outcome events or summary measures over time	p. 11
Main results	16	(*a*) Give unadjusted estimates and, if applicable, confounder-adjusted estimates and their precision (eg, 95% confidence interval). Make clear which confounders were adjusted for and why they were included	Table 2, p. 7
		(*b*) Report category boundaries when continuous variables were categorized	p. 6, 9
		(*c*) If relevant, consider translating estimates of relative risk into absolute risk for a meaningful time period	-
Other analyses	17	Report other analyses done—eg analyses of subgroups and interactions, and sensitivity analyses	p. 11
**Discussion**			
Key results	18	Summarise key results with reference to study objectives	p. 12
Limitations	19	Discuss limitations of the study, taking into account sources of potential bias or imprecision. Discuss both direction and magnitude of any potential bias	p. 12
Interpretation	20	Give a cautious overall interpretation of results considering objectives, limitations, multiplicity of analyses, results from similar studies, and other relevant evidence	p. 13-16
Generalisability	21	Discuss the generalisability (external validity) of the study results	p. 15
**Other information**			
Funding	22	Give the source of funding and the role of the funders for the present study and, if applicable, for the original study on which the present article is based	

*Give information separately for exposed and unexposed groups.

Note: An Explanation and Elaboration article discusses each checklist item and gives methodological background and published examples of transparent reporting. The STROBE checklist is best used in conjunction with this article (freely available on the Web sites of PLoS Medicine at http://www.plosmedicine.org/, Annals of Internal Medicine at http://www.annals.org/, and Epidemiology at http://www.epidem.com/). Information on the STROBE Initiative is available at http://www.strobe-statement.org.

For peer review only
